# Enhancing the catalytic activity of hydronium ions through constrained environments

**DOI:** 10.1038/ncomms14113

**Published:** 2017-03-02

**Authors:** Yuanshuai Liu, Aleksei Vjunov, Hui Shi, Sebastian Eckstein, Donald M. Camaioni, Donghai Mei, Eszter Baráth, Johannes A. Lercher

**Affiliations:** 1 Department of Chemistry and Catalysis Research Center, TU München, Lichtenbergstrasse 4, 85748 Garching, Germany; 2 Pacific Northwest National Laboratory, Institute for Integrated Catalysis, P.O. Box 999, Richland, Washington 99352, USA

## Abstract

The dehydration of alcohols is involved in many organic conversions but has to overcome high free-energy barriers in water. Here we demonstrate that hydronium ions confined in the nanopores of zeolite HBEA catalyse aqueous phase dehydration of cyclohexanol at a rate significantly higher than hydronium ions in water. This rate enhancement is not related to a shift in mechanism; for both cases, the dehydration of cyclohexanol occurs via an E1 mechanism with the cleavage of C_β_–H bond being rate determining. The higher activity of hydronium ions in zeolites is caused by the enhanced association between the hydronium ion and the alcohol, as well as a higher intrinsic rate constant in the constrained environments compared with water. The higher rate constant is caused by a greater entropy of activation rather than a lower enthalpy of activation. These insights should allow us to understand and predict similar processes in confined spaces.

## Abstract (web summary)

Alcohol dehydration can be challenging in aqueous phase. Here the authors show that hydronium ions confined with zeolite pores catalyse alcohol dehydration at a significantly increased rate relative to aqueous phase hydronium ions, driven by an increased association between the ion and alcohol and a greater entropy of activation.

Despite the seemingly ubiquitous use in organic conversion sequences, the dehydration of alcohols by hydronium ions in aqueous phase is surprisingly challenging, requiring reaction temperatures above 100 °C to occur at industrially acceptable rates^1^. The reasons for this lie in significant enthalpic and entropic barriers for the formation of carbocationic intermediates and for their decomposition to form the olefin and water. Enzymes, in contrast, are able to catalyse dehydration of alcohols with high rates at temperatures close to ambient^2^, which is attributed to the unique microenvironment of the catalytically active centers in the three-dimensional enzyme structures and the nearly concerted acid base interactions. In translating this concept to inorganic catalysts we have shown in recent preliminary experiments that zeolite pores are able to substantially increase the rate at which hydronium ions catalyse reactions^3^.

To delineate the thermodynamic and kinetic impact of the sub-nanometre-sized confines on the catalytic chemistry of hydronium ions, the kinetics and elementary steps of the dehydration of a secondary alcohol, cyclohexanol, in water and in pores of zeolite Beta (BEA) are explored. In-depth characterizations of this zeolite by extended X-ray absorption fine structure and ^27^Al magic angle spinning nuclear magnetic resonance (NMR) spectroscopy showed that in the presence of adsorbed water the charge-balancing protons form hydronium ions, H_3_O^+^(H_2_O)_*n*_, which reside locally near the zeolite Al^3+^ T-site bearing the charge-balancing protons in the absence of water^4^,^5^,^6^. Previous studies also provided infrared spectroscopic evidence for the formation of H-bonded and protonated polar molecules (for example, alcohol and water) at acid sites on HBEA and HZSM-5 zeolites^7^,^8^. More importantly, the principal reaction network of the zeolite BEA-catalysed dehydration established by *in situ* magic angle spinning ^13^C NMR spectroscopy^9^ in aqueous phase enables us to analyse in this contribution the role of the confines on the catalytic properties of hydronium ions.

Here, thermochemical and kinetic measurements are used in conjunction with density functional theory (DFT) and isotope labelling, to elucidate quantitatively the reaction pathway in the aqueous-phase dehydration of alcohols in constrained environment and analyse the benefits of such a sterically tailored environment based on transition state theory.

## Results

### H_3_PO_4_-catalysed aqueous-phase cyclohexanol dehydration

Dehydration of cyclohexanol catalysed by dilute hydronium ions (dissociated from H_3_PO_4_) leads solely to the formation of cyclohexene. Possible alkylation products, cyclohexyl cyclohexene and dicyclohexyl ether, were not observed. The absence of bimolecular reactions is concluded to be caused by the unfavourable conditions for bimolecular reactions at the low reactant concentrations. The low solubility of cyclohexene in the aqueous phase also disfavours bimolecular reactions with reactive intermediates such as cyclohexyloxonium and cyclohexyl cations.

The concentration of the hydronium ions, on proper corrections (Supplementary Table 1), has been used to calculate the turnover frequencies (TOFs) reported in Table 1 for the H_3_PO_4_-catalysed dehydration (Supplementary Fig. 1). The rate of the cyclohexanol dehydration was proportional to the concentration of hydronium ions, rather than the total H_3_PO_4_ concentrations, consistent with specific acid catalysis in the studied range of dilute H_3_PO_4_ concentrations (0.02–0.09 M; Supplementary Fig. 2). The turnover rate of cyclohexanol dehydration is roughly first order in alcohol at low concentrations (∼0.1–0.3 M), but deviates from first-order behaviour at higher concentrations (0.90 M; Supplementary Fig. 3). The measured activation barrier was ∼158 kJ mol^−1^ at two alcohol concentrations (0.32 and 0.90 M; see Table 1 and Supplementary Fig. 4).

### Zeolite-catalysed aqueous-phase cyclohexanol dehydration

The detailed physicochemical properties of zeolite HBEA150 (SiO_2_/Al_2_O_3_=150) are given in the Supplementary Information (Supplementary Figs 5 and 6, and Supplementary Tables 2 and 3). As with H_3_PO_4_, cyclohexene was the main product of cyclohexanol dehydration on zeolite HBEA in dilute aqueous solutions (0.32–1.1 M). The nearly 100% selectivity to cyclohexene at short reaction times (for example, <1 h at 200 °C) indicates that water elimination proceeds preferentially via an intramolecular rather than an intermolecular pathway. In contrast to H_3_PO_4_, HBEA catalysed also ether formation and C–C alkylation reactions at higher conversions^9^, suggesting that the large intracrystalline voids of zeolite BEA allow bimolecular reactions^10^.

The rates and TOFs for the dehydration of cyclohexanol on HBEA (Supplementary Fig. 7) are also reported in Table 1. The dehydration TOFs on HBEA were an order of magnitude higher than those catalysed by aqueous-phase hydronium ions at 0.32 M alcohol concentration (Table 1). It is noteworthy that TOFs were obtained by normalizing the rates to the concentration of total Brønsted acidic sites (BAS) in HBEA150 (Supplementary Table 2), as we have shown earlier that all the BAS are present in the form of solvated hydronium ions^4^, which are equally active in aqueous-phase dehydration^11^. Surprisingly, the activation energies (162–164 kJ mol^−1^, see Supplementary Fig. 8) measured on HBEA in aqueous phase were similar to those (158 kJ mol^−1^) measured in aqueous H_3_PO_4_. However, the rate was zero-order in cyclohexanol (measured: 0.1±0.1; see Supplementary Fig. 9), much lower than the first-order dependence observed in H_3_PO_4_ solution. The zero-order kinetics for cyclohexanol suggests that nearly all hydronium ions are interacting with the alcohol or maintain—at least—a fully occupied precursor state to the alcohol-hydronium ion complex. Another interesting observation is that the dehydration of cyclohexanol catalysed by a mixture of H_3_PO_4_ (0.02 M) and HBEA150 (140 mg) showed a significantly higher reaction rate than the sum of the individual rates on each acid (Supplementary Table 4).

### Adsorption of cyclohexanol on zeolite HBEA

**Figure 1:**
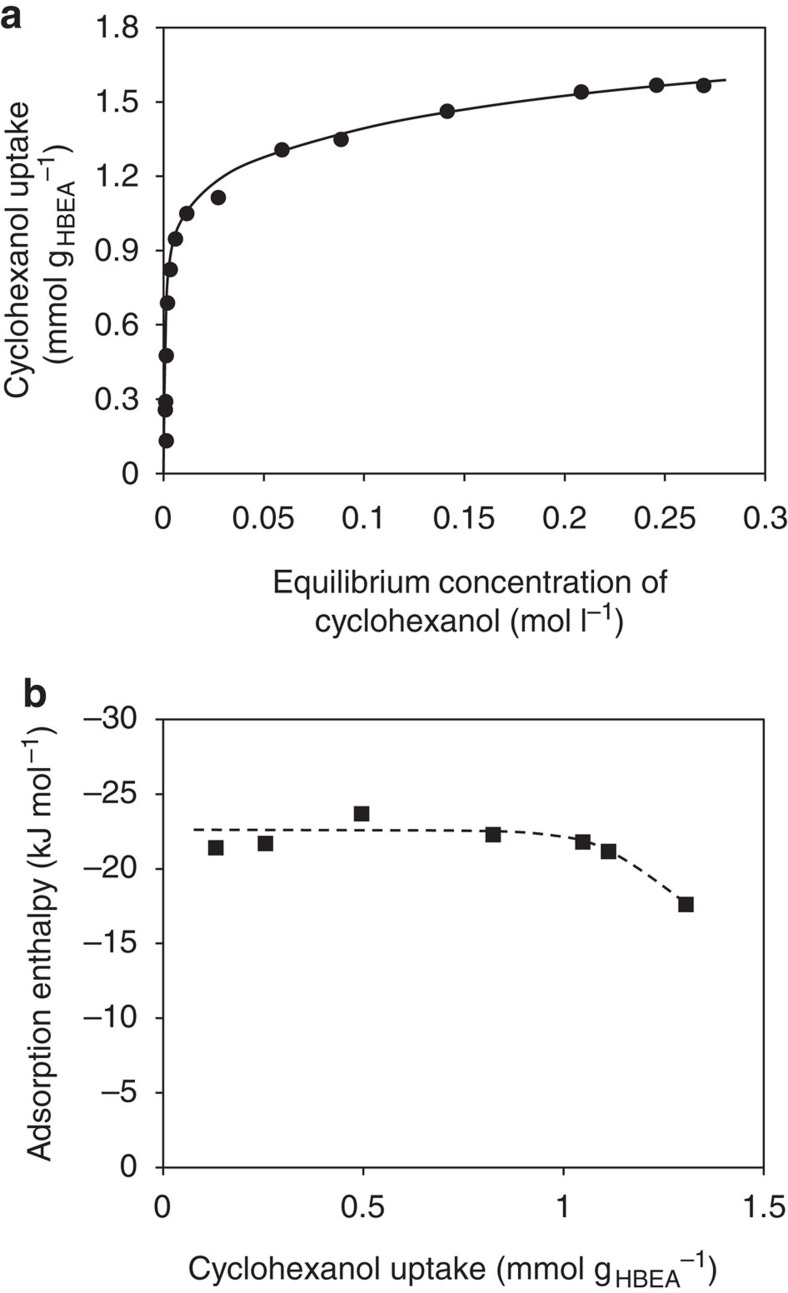
Adsorption of cyclohexanol from aqueous solutions onto HBEA. (**a**) Cyclohexanol adsorption isotherm measured by ^1^H NMR and (**b**) heat of adsorption measured by calorimetry, both determined for aqueous solutions and HBEA150 at 25 °C.

The adsorption isotherm of cyclohexanol and the associated heats of adsorption are shown in Fig. 1. Microgravimetric analyses of gas-phase cyclohexanol adsorption on the zeolite provide an estimate of the maximum alcohol uptake in the absence of water (see Supplementary Fig. 10).

Langmuir-type isotherms satisfactorily describe the uptake of cyclohexanol from both gas (without water) and aqueous phase on zeolite HBEA150. The saturation uptakes of cyclohexanol and water (Supplementary Table 5) correspond to eight cyclohexanol and ten water molecules per unit cell at the saturation limit (room temperature). In good agreement, a maximum of 8 cyclohexanol molecules or 20–30 water molecules in 1 unit cell are allowable at the highest pore filling degree, according to DFT calculations.

Adsorption equilibrium constants (*K*_ads_) for cyclohexanol uptake along with the measured enthalpies and entropies were determined (Supplementary Table 6). The molar adsorption enthalpy of cyclohexanol adsorbed from aqueous phase is −22 kJ mol^−1^ (Fig. 1). This enthalpy is the result of transferring cyclohexanol from the aqueous medium (breaking H-bonding between cyclohexanol and water) and the displacement of water by cyclohexanol in the zeolite pores, the magnitude depending on the strengths of interactions between cyclohexanol/water and the pores, as well as the BAS.

With increasing temperatures (7–80 °C), saturation uptakes decreased from 1.75 to 1.31 mmol g_HBEA_^−1^ (Supplementary Table 5). This decrease is caused by the density change of the adsorbed phase in the micropore with temperature^12^. By extrapolating saturation uptakes to 160–200 °C, it is estimated that ∼5 cyclohexanol molecules are present per unit cell at saturation limit under reaction conditions (see Supplementary Note 1). Assuming that the remaining micropore volume is filled by water, the concentration of water molecules in the pore would increase to ∼20 per unit cell at reaction temperatures. It is noteworthy that the initial alcohol-to-water ratio in the zeolite pore is, thus, a factor of 50 higher than in solution (for example, 5.3–5.6 × 10^−3^ for 0.32 M solution at 160–200 °C). Extrapolation of *K*_ads_ to reaction temperatures suggests that *K*_ads_ decreased from 20 to 12 as the reaction temperature increased from 160 to 200 °C. This suggests an almost complete pore filling under reaction conditions, in line with the zero-order kinetic regime for the main dehydration pathway.

### Mechanism of dehydration of cyclohexanol in aqueous phase

Having established the principal kinetic features of the elimination of water from cyclohexanol catalysed by hydronium ions, we use the H/D kinetic isotope effect (KIE) and ^18^O-tracer experiments, to investigate whether the elimination occurs via an E1 or E2 mechanism.

The TOFs for dehydration using C_6_H_11_OH and C_6_D_11_OD (forming C_6_D_11_OH on exchange with H_2_O) are shown in Table 2. H/D KIEs of ∼3 were observed for olefin formation catalysed by hydronium ions in open water and in the nanopores of HBEA. A KIE of such a magnitude indicates that C–H(*D*) bond cleavage is involved in the kinetically relevant step (that is, its rate constant appears in the kinetic expression). The primary KIE is inconsistent with the formation of the carbocation or the C–O bond cleavage being rate determining. Both steps would have secondary KIEs for rehybridization of α-C from *sp*^3^ to *sp*^2^, estimated to be <1.3 at 150–190 °C. In turn, this indicates that either an E1 mechanism with a kinetically relevant C–H bond cleavage or an E2 mechanism in which the C–O and the C–H bonds are cleaved in a concerted step is in agreement with the observed KIE.

To discriminate between the two mechanistic possibilities, ^18^O-labelled water was used as the solvent (Table 3). The reverse rate at 20% conversion, that is, the hydration of cyclohexene, would lead to ∼2% ^18^O incorporation, based on the analysis of the effective equilibrium constant (Supplementary Table 7) obtained by fitting the derived rate expression (details shown in Supplementary Note 2) to the *in situ* time-resolved infrared data collected during cyclohexanol dehydration (Supplementary Fig. 11). As olefin hydration hardly occurred under the applied conditions on HBEA and H_3_PO_4_, the E2-like pathways alone, with concerted C–O and C–H bond scissions, cannot explain the significant ^18^O incorporation (9–17%) into cyclohexanol. With the *S*_N_2 path for oxygen exchange between water and secondary/tertiary alcohols also ruled out^13^,^14^,^15^,^16^, the only possible pathway for this level of ^18^O incorporation would be recombination between ^18^O water and an intermediate, which is formed on C–O bond cleavage and which precedes the C_β_–H bond cleavage TS. This, in turn, makes the E1-type path the dominating mechanism for dehydration of cyclohexanol, regardless of whether the hydronium ion exists in homogeneous solution or localized in a pore.

### DFT calculations of hydronium ion catalysed pathways in HBEA

**Figure 2:**
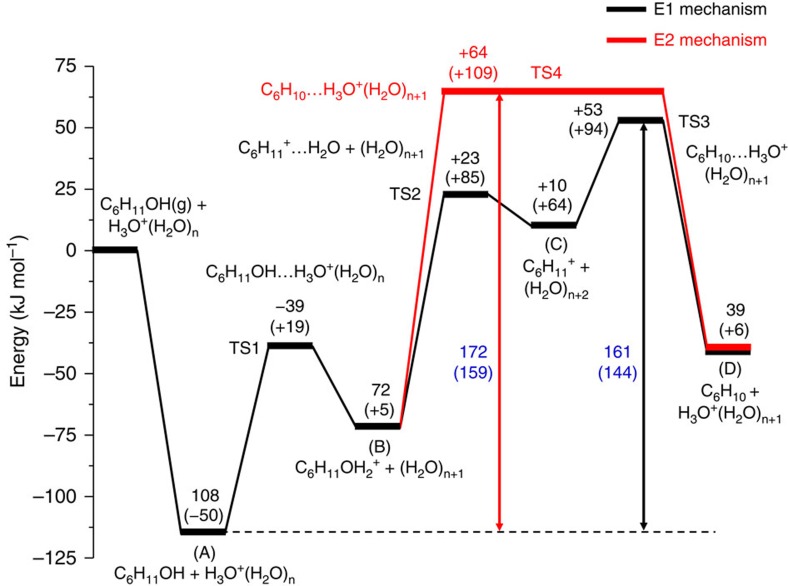
DFT calculations of cyclohexanol dehydration on HBEA. The energy diagram is shown for the aqueous-phase dehydration of cyclohexanol over a periodic HBEA (Al_4_H_4_Si_60_O_128_) model. The active site in zeolite equilibrated with aqueous phase is modelled by H_3_O^+^(H_2_O)_7_, with the configurations and energies optimized. All species, except for those denoted with (g), are in the unit cell. The detailed structures and configurations of the adsorbed intermediates, transition states and the H_3_O^+^(H_2_O)_7_ hydronium ion cluster are shown in the Supplementary Fig. 13. Enthalpy and free energy values (at 170 °C) are shown outside and inside the brackets, respectively.

The DFT calculations only address the kinetically relevant intermediates for protonation and H_2_O elimination. Other steps, such as desorption of water and olefins, will be discussed elsewhere, because they are irrelevant for the rates of dehydration. The calculated energy profiles for the reaction at 170 °C are shown in Fig. 2. The BEA unit cell may contain three to ten H_2_O molecules in proximity to the hydronium ion. For the theoretical evaluation of the interaction of the alcohol with the hydronium ion, we chose an example hydronium ion cluster with a H_3_O^+^(H_2_O)_7_ structure, the presence of which was identified by *ab initio* molecular dynamics simulations (a total of 26 water molecules in the unit cell; see Supplementary Fig. 12). This structure includes extended hydration shells beyond the first shell.

Up to four cyclohexanol molecules were considered in addition to the hydronium ion in one BEA unit cell. The alcohol is seen to interact with the hydronium ion, forming an H-bond, while also interacting with the pore walls. The calculated enthalpy and free energy for cyclohexanol (gas) adsorption and subsequent interaction with the zeolitic hydronium ion (A, Fig. 2) were −108 and −50 kJ mol^−1^, respectively. These values are in reasonable agreement with gas phase adsorption and calorimetric measurements (Supplementary Fig. 10). The H-bonded cyclohexanol is protonated and forms an alkoxonium ion (B, Fig. 2). The activation barrier for this step is 69 kJ mol^−1^ (from A to TS1, Fig. 2). This protonation step is endothermic (Δ*H*°=+ 36 kJ mol^−1^) and endergonic (Δ*G*°=+55 kJ mol^−1^). Thus, the protonated alcohol is expected to be a minority species at typical reaction temperatures.

For comparison DFT calculations were performed for both E1- and E2-type elimination paths. On the E1-type path, the C–O bond cleavage has an activation barrier of 95 kJ mol^−1^, with an entropy gain of 34 J mol^−1^ K^−1^. In TS2, the leaving OH_2_ is almost neutral and the positive charge remains largely on the [C_6_H_11_] moiety. Next, the C_6_H_11_^+^ carbenium ion deprotonates to the hydronium ion cluster forming cyclohexene. In TS3, a H_2_O molecule nearby acts as the base to abstract the β-H; the C_β_–H bond is almost fully broken (2.46 Å; see Supplementary Fig. 13). This deprotonation has a small barrier (43 kJ mol^−1^) in the forward direction and a higher barrier (92 kJ mol^−1^) in the reverse direction. The higher free-energy barrier for deprotonation (from C to TS3) than for C–O bond recombination (from C to TS2) is in line with the kinetic relevance of C–H bond cleavage concluded from the measured primary H/D isotope effects.

In comparison, on the E2-type path, the enthalpy of activation and entropy of activation calculated at 170 °C were 137 kJ mol^−1^ and 74 J mol^−1^ K^−1^, respectively (from B to TS4). These activation energies and entropies are larger than the corresponding values for the E1-type path (Fig. 2), making the latter also more plausible from the point of DFT modeling.

### Causes for the rate increase by pore constraint

Let us analyse in the next step the reasons for the markedly higher (for example, ∼16 times at 180 °C and 0.32 M cyclohexanol) rates catalysed by hydronium ions present in the pore of zeolite BEA compared with that in open water.

For brevity, in aqueous H_3_PO_4_, we represent the hydrated hydronium ion as an Eigen-type^17^,^18^ structure, H_3_O^+^(H_2_O)_3_(aq), in which only the numbers of first-shell waters are shown. Without steric constraints, the reaction starts with the association of the hydronium ion with cyclohexanol, presumably replacing a H_2_O molecule by cyclohexanol in the first solvation shell of the hydronium ion^19^ (equation (1)).


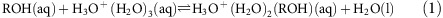


Under reaction conditions, this step is quasi-equilibrated, with an association constant *K*_L,a_ (where the subscript ‘L' stands for the liquid phase and ‘a' stands for association).

The steps following the association of the proton with the alcohol are all unimolecular, as we demonstrate later. Together, they can be written as equation (2), with a collective forward rate constant *k*_L,d_ (where ‘d' stands for dehydration)


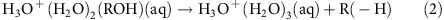


where R(−H) represents the olefin product (cyclohexene) having one less hydrogen than the alkyl group R (cyclohexyl).

The rate of dehydration normalized to the concentration of total hydronium ions [H_3_O^+^]_0_ ([H_3_O^+^]_0_=[H_3_O^+^(H_2_O)_3_]+[H_3_O^+^(H_2_O)_2_ROH]) is TOF (Table 1) and defined as the product of the rate constant *k*_L,d_ and the fraction of hydronium ions associated with the alcohol, *θ*_L,a_. (equations (3) and (4); details of derivation and calculation shown in Supplementary Note 3 and 4)


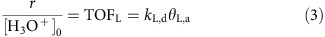



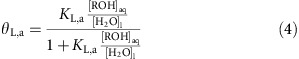


The association constant *K*_L,a_ was derived from initial reaction rates, *r*, measured at two different alcohol concentrations (0.32 and 0.90 M). The values of *K*_L,a_, the alcohol-hydronium ion association equilibrium constant, decreased modestly from 40 to 37 with increasing temperature from 160 to 200 °C (Supplementary Table 8). A similar weak temperature dependence had been reported for the protonation of C_2_–C_4_ aliphatic alcohols by aqueous sulfuric acids (exothermicity of −2 kJ mol^−1^)^20^. At 0.32 M and 160–200 °C, the fraction of hydronium ions associated with cyclohexanol (*θ*_L,a_) was ∼0.17 (Supplementary Table 8). With the regressed *K*_L,a_ and *k*_L,d_, the changes in enthalpy and entropy for association equilibrium between hydronium ion and cyclohexanol in H_3_PO_4_, as well as the intrinsic activation barriers for H_3_PO_4_-catalysed dehydration were determined (Supplementary Figs 14 and 15).

In analogy to the plain aqueous-phase dehydration, the rate normalized to the hydronium ion concentration in zeolite HBEA (TOF) is


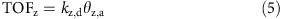


*θ*_z,a_ is the fractional coverage or association of the hydronium ions with cyclohexanol. In zeolite HBEA, ∼5 cyclohexanol and ∼20 water molecules occupy a unit cell, whereas in a 0.32 M solution, 1 cyclohexanol molecule shares the volume with 180 water molecules. Consequently, *θ*_z,a_ has a value at least close to 1, in comparison with a *θ*_L,a_ value of 0.17 in a solution containing 0.02 M H_3_PO_4_ and 0.32 M cyclohexanol. In turn, the rate constant in zeolite HBEA (*k*_z,d_) is at least ∼2.7 times higher than that in the homogeneous acid solution (*k*_L,d_) (see Supplementary Note 5). Altogether, the analysis shows that the HBEA pore provides an environment that not only increases the fraction of hydronium ions associated with alcohol, but also increases the intrinsic dehydration rate constant, collectively contributing to more than one order of magnitude enhancement in rate compared to the homogeneously catalysed dehydration (Table 1).

Interestingly, the observed rates with a mixture of H_3_PO_4_ and HBEA were higher than the sum of rates obtained with the individual acids (Supplementary Table 4), presumably due to phosphoric acid being adsorbed in the pore^21^,^22^. We speculate that additional hydronium ions generated by dissociation of phosphoric acid in the pore partly account for this rate enhancement, whereas alternative elimination pathways (for example, cyclohexyl phosphate ester mediated^23^) may be available in the unique confines of the zeolite (see extended discussion in the Supplementary Note 6). However, the concentration of H_3_PO_4_ and the extent of its dissociation in the zeolite pore at reaction temperature are presently not known, preventing a quantitative analysis of the potential causes.

## Discussion

In the catalytic sequence of the zeolite-catalysed dehydration, cyclohexanol is first adsorbed from aqueous solution into intracrystalline voids. From aqueous phase, this step is accompanied with a change of −22 kJ mol^−1^ in enthalpy and −25 J mol^−1^ K^−1^ in entropy (Supplementary Table 6). In the presence of water, the zeolite BAS form confined hydronium ions^4^,^5^,^6^,^7^,^8^. The hydronium ion protonates the alcohol, to which it is H-bonded. DFT calculations suggest that the alcohol protonation equilibrium constant in zeolites depends critically on the number of water molecules in the hydronium-ion cluster (Supplementary Table 9). Although water has a smaller proton affinity than cyclohexanol, a cluster of water molecules (*n*≥3) may have a higher proton affinity than cyclohexanol. As a consequence, proton transfer from a hydronium ion-water cluster to cyclohexanol will become progressively more favourable as the cluster decreases in size. In aqueous solution, the prevalent hydronium ion in zeolite HBEA was simulated as H_3_O^+^(H_2_O)_7_. With this cluster, protonation of cyclohexanol is thermodynamically unfavourable (DFT: Δ*G*°=+55 kJ mol^−1^). Accordingly, a majority of the BAS interacts with the alcohol without a significant extent of proton transfer. In turn, the measured enthalpy of activation (159 kJ mol^−1^) and corresponding entropy change (87 J mol^−1^ K^−1^) reflect the difference between the kinetically relevant TS (that is, C_β_–H bond cleavage TS) and the H-bonded alcohol state (A in Fig. 2).

Because of the weak temperature dependence of *K*_L,a_ and [ROH]_aq_/[H_2_O]_l_ ratio (equation (3)), the intrinsic activation barrier (Table 4) is anticipated to be close to the measured energy of activation (Table 1). As discussed, the intrinsic rate constants for H_3_PO_4_-catalysed dehydration were determined at 160–200 °C, yielding the activation enthalpy (157 kJ mol^−1^) and the activation entropy (73 J mol^−1^ K^−1^). Thus, the dehydration of aqueous cyclohexanol occurs in HBEA with a similar activation enthalpy, yet a greater entropy gain than in aqueous acidic solution (Table 4).

Thus, cyclohexanol dehydration was catalysed with markedly higher rates when the hydronium ions were confined in zeolite pores. This rate enhancement is partly explained by the intrinsic rate constant for dehydration, which was at least two to three times higher in HBEA than in water. It is noteworthy that the intrinsic enthalpies of activation were similar for catalysis in BEA pores as in water, whereas the associated entropy of activation was greater for hydronium ion catalysis in the zeolite pores than in water. The largest effect arising from a constrained environment is, however, related to the higher association extent of cyclohexanol with the hydronium ion. We attribute this to the lower entropy loss when forming an association complex in the zeolite pore. In contrast to plain aqueous phase, the lower entropy of molecules mobile in pores of molecular sieves will lead to a much smaller loss in forming the reactant-catalyst adduct. Such enhanced association between substrate and active site, as well as the entropically favoured intrinsic kinetics within sterically constrained environments bears a strong resemblance to enzyme catalysis^24^,^25^. This work suggests a new approach to designing reaction environments that could lead to enzyme-like activities and selectivities.

## Methods

### Zeolite catalysts

Zeolite HBEA150 (SiO_2_/Al_2_O_3_=150) was obtained from Clariant in H-form. HBEA150 was calcined at 500 °C in a 100 ml min^−1^ flow of dry air for 6 h before the reaction. Detailed descriptions of characterization methods are provided in the Supplementary Methods.

### Liquid-phase adsorption and calorimetry

Heat of adsorption, that is, uptake of cyclohexanol (Sigma-Aldrich, 99%) from aqueous solutions into zeolite HBEA150, was determined by liquid calorimetry using a Setaram Calvet C80 calorimeter. Reversal mixing cells were used, to separate the adsorptive from the adsorbent. The lower compartment was loaded with 0.03 g zeolite (*m*) immersed in 0.8 ml water. The upper compartment was loaded with 0.2 ml of the desired cyclohexanol solution resulting in a total volume (*V*) of 1 ml with a concentration *c*_0_. Reference cell is loaded with identical compositions, without zeolite. Uptake (*q*) was determined using liquid NMR and quantification was accomplished adding an internal standard (1,3,5-trioxane; Sigma-Aldrich, ≥99%) to the solution at equilibrium (*c*_e_), assuming *q*=*V*(*c*_0_−*c*_e_)*m*^−1^. Adsorption isotherms were obtained immersing 100, 50 or 20 mg of zeolite in a cyclohexanol solution of a defined concentration for at least 24 h. The solution was separated from the zeolite and the residual concentration was determined via liquid NMR using the internal standard, trioxane.

### Kinetic measurements

Kinetic measurements were performed at 160–200 °C using a 300 ml Hastelloy PARR reactor. An example of a typical reaction in aqueous phase: 3.3 g cyclohexanol and 100 ml 0.02 M aqueous H_3_PO_4_ (Sigma-Aldrich, ≥99.999% trace metals basis) solution or 140 mg HBEA and 80 ml 0.32 M aqueous cyclohexanol solution are sealed in the reactor. In all cases, the reactor is then pressurized with 50 bar H_2_ at room temperature and heated up while stirred vigorously (∼700 r.p.m.). Rates do not vary with the stirring speed that is >400 r.p.m. (Supplementary Fig. 16). The reaction time is reported counting from the point when the set temperature is reached (12–15 min). On completion, the reactor is cooled using an ice/water mixture. As olefin is formed, which is segregated as another liquid phase, the contents are extracted using dichloromethane (Sigma-Aldrich, HPLC grade; 25 ml per extraction, 4 times) or ethyl acetate. It is important that the extraction work-up be completed in a short period of time (20 min), to minimize the loss of the volatile olefin phase; this way, the carbon balance could be maintained typically better than 85% and even better than 95% in favourable cases. The organic phase after being dried over sodium sulfate (Acros Organics, 99%, anhydrous) is analysed on an Agilent 7890A GC equipped with a HP-5MS 25 m × 0.25 μm (i.d.) column, coupled with Agilent 5975C MS. Then, 1,3-dimethoxybenzene (Sigma-Aldrich, 99%) was used as the internal standard for quantification.

### H/D KIEs and ^18^O tracer experiments

Rates of dehydration of perdeuterated cyclohexanol (0.10–0.11 M; present as C_6_D_11_OH in water) were measured in the Parr reactor, using protocols identical to those described for standard reactions using non-labelled alcohol (see above).

Experiments using ^18^O-labelled water and non-labelled cyclohexanol (0.3 M) were carried out in a ∼2 ml stirred batch reactor constructed from a stainless steel ‘tee' (HiP), whereas ensuring similar solution-to-headspace ratios (0.3–0.4) as in the Parr reactor. The mixture after reaction was extracted with dichloromethane (0.5 ml per extraction, 4 times), dried over Na_2_SO_4_ and analysed with gas chromatography–mass spectrometry. The intensity ratio between two O-containing fragment ions (*m*/*e*=57 and 59) can be used to quantify the extent of ^18^O incorporation into cyclohexanol (the ratio between the single ion areas for *m*/*e*=59 and *m*/*e*=57 is 0.01 for unlabelled alcohol).

### DFT calculations

All DFT calculations employed a mixed Gaussian and plane wave basis sets and were performed using the CP2K code^26^. The basis set superimposition error derived from Gaussian localized basis set used in our CP2K calculations has been estimated to be ∼3 kJ mol^−1^ (ref. 27). The core electrons were represented by norm-conserving Goedecker–Teter–Hutter pseudo-potentials^28^,^29^,^30^ and the valence electron wave function was expanded in a double-zeta basis set with polarization functions^31^ along with an auxiliary plane wave basis set with an energy cutoff of 360 eV. In all calculations we used the generalized gradient approximation exchange-correlation functional of Perdew, Burke and Enzerhof^32^. All configurations were optimized using the Broyden–Fletcher–Goldfarb–Shanno algorithm with self-consistent field (SCF) convergence criteria of 10^−8^ a.u. To compensate the long-range van der Waals interaction between adsorbate molecules and the zeolite, we employed the DFT-D3 scheme^33^ with an empirical damped potential term added into the energies obtained from exchange-correlation functional. A periodic three-dimensional all siliceous BEA structure of Si_64_O_128_ with experimental lattice parameters of 12.6614 × 12.6614 × 26.4061 Å^3^ was used in this work^34^. The unit cell of the HBEA with Si/Al=15 ratio then was built by simply replacing four T-site (T3, T4, T5 and T9) Si atoms with four Al atoms. This resulting negative charges were compensated by adding four H atoms at the oxygen atoms, which are close neighbours of Al atoms on the zeolite frame, yielding the active BAS, that is, Si-O(H)-Al-O of the HBEA zeolite.

The adsorption energy of cyclohexanol into the pore of HBEA zeolite is calculated as follows:


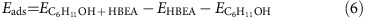


where 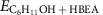 is the total energy of cyclohexanol adsorbed in the pore of HBEA, *E*_HBEA_ is the total energy of the HBEA, and 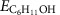 is the total energy of cyclohexanol in vacuum.

The Gibbs free energy changes (Δ*G*°) along different reaction pathways were calculated using statistical thermodynamics^35^. To account for important entropic contribution, the method for calculating the vibrational entropic term, employed by De Moor *et al*.^36^, was used in this work.

### Data availability

All data are available within the article and its Supplementary Information files, and from the authors upon reasonable request.

## Additional information

**How to cite this article:** Liu, Y. *et al*. Enhancing the catalytic activity of hydronium ions through constrained environments. *Nat. Commun.* **8,** 14113 doi: 10.1038/ncomms14113 (2017).

**Publisher's note:** Springer Nature remains neutral with regard to jurisdictional claims in published maps and institutional affiliations.

## Supplementary Material

Supplementary Information Supplementary figures, supplementary tables, supplementary notes, supplementary methods and supplementary references.

## Figures and Tables

**Table 1:** Rates and activation energies for dehydration of cyclohexanol.

Reaction conditions*	**Kinetic measurement**†	**Reaction temperature (°C)**					***E***_**a**_‡ **(kJ** **mol**^**−1**^**)**
		**160**	**170**	**180**	**190**	**200**	
Cyclohexanol (∼0.32 M), 0.02 M H_3_PO_4_	Rate (mol l^−1^ s^−1^)	5.5 × 10^−6^	1.3 × 10^−5^	2.9 × 10^−5^	6.4 × 10^−5^	1.5 × 10^−4^	157±3
	TOF (mol_alcohol_ mol_acid sites_^−1^ s^−1^)	1.4 × 10^−3^	3.5 × 10^−3^	8.6 × 10^−3^	2.1 × 10^−2^	5.6 × 10^−2^	
Cyclohexanol (∼0.90 M), 0.02 M H_3_PO_4_	Rate (mol l^−1^ s^−1^)	1.3 × 10^−5^	3.1 × 10^−5^	6.9 × 10^−5^	1.5 × 10^−4^	3.7 × 10^−4^	158±4
	TOF (mol_alcohol_ mol_acid sites_^−1^ s^−1^)	2.9 × 10^−3^	7.6 × 10^−3^	1.9 × 10^−2^	4.4 × 10^−2^	1.2 × 10^−1^	
Cyclohexanol (∼0.32 M), 140 mg HBEA150	Rate (mol g_HBEA_^−1^ s^−1^)	3.7 × 10^−6^	1.0 × 10^−5^	2.6 × 10^−5^	6.4 × 10^−5^	1.8 × 10^−4^	164±3
	TOF (mol_alcohol_ mol_acid sites_^−1^ s^−1^)	1.9 × 10^−2^	5.2 × 10^−2^	1.4 × 10^−1^	3.3 × 10^−1^	9.3 × 10^−1^	
Cyclohexanol (∼0.90 M), 140 mg HBEA150	Rate (mol g_HBEA_^−1^ s^−1^)	4.2 × 10^−6^	1.2 × 10^−5^	3.4 × 10^−5^	7.2 × 10^−5^	2.0 × 10^−4^	162±4
	TOF (mol_alcohol_ mol_acid sites_^−1^ s^−1^)	2.2 × 10^−2^	6.2 × 10^−2^	1.8 × 10^−1^	3.8 × 10^−1^	1.03	

^*^Reactor was pressurized with 50 bar H_2_ at ambient temperature and stirred vigorously at 700 r.p.m. The rates were determined from the formation of cyclohexene after the set temperature was reached. The concentrations denoted are based on the density of water at room temperature.

^†^Turnover frequency (TOF) is determined as olefin formation rate (mol l^−1^ s^−1^) normalized to the concentration of hydronium ions (H_3_PO_4_) or total BAS (HBEA). The concentration of hydronium ions in the H_3_PO_4_-catalysed experiments depends on temperature and cyclohexanol concentration.

^‡^Activation barriers are determined from the Arrhenius plots for TOFs (a directly measured property).

**Table 2:** H/D isotope effects*.

**Reactant**	**TOF**	
	**H**_**3**_**PO**_**4**_ **(10**^**−3**^ **s**^**−1**^**)**†	**HBEA (10**^**−2**^ **s**^**−1**^**)**‡
C_6_H_11_OH	3.5±0.2	5.5±0.3
C_6_D_11_OD§	1.2±0.1	1.9±0.1
KIE	3.0±0.4	2.9±0.3

KIE, kinetic isotope effect; TOF, turnover frequency.

^*^Reactant conversions were kept at 5–10% and dicyclohexyl ether selectivities at 0–2%; cyclohexanol and perdeuterated cyclohexanol were dissolved in unlabelled water (∼0.1 M); 98 atom% isotopic purity for C_6_D_11_OD.

^†^At 180 °C.

^‡^At 170 °C.

^§^Forming C_6_D_11_OH on exchange with H_2_O.

**Table 3:** ^18^O exchange during cyclohexanol dehydration*.

**Catalyst**	^**18**^**O in the recovered alcohol (%)**	**Conversion (%)**
HBEA	9	19
H_3_PO_4_	17	18

^*^Extent of ^18^O exchange from H_2_^18^O (97% isotopic purity) into cyclohexanol during dehydration of unlabelled cyclohexanol (0.30 M in H_2_^18^O) over HBEA and H_3_PO_4_ in aqueous phase at 180 °C.

**Table 4:** Intrinsic activation parameters for aqueous phase dehydration of cyclohexanol*.

**Kinetic parameter**	**H**_**3**_**PO**_**4**_	**HBEA**
Δ*H*°^‡^ /kJ mol^−1^	157±3	159±4
Δ*S*°^‡^/J mol^−1^ K^−1^	73±7	87±9
Δ*G*°^‡^_180_/kJ mol^−1^	124±1	120±1

^*^Intrinsic standard activation enthalpies, entropies and Gibbs free energies are determined according to transition state theory, see Supplementary Note 7. The error bars for Δ*H*°^‡^ and Δ*S*°^‡^ represent the 1−*σ* s.d., whereas the error bar for Δ*G*°^‡^ represents the maximum error rounded up to the nearest integer.
