# Supplementary material for: Enhancing the catalytic activity of hydronium ions through constrained environments
Source: Nat Commun. 2017 Mar 2;8:14113. doi: 10.1038/ncomms14113 (PMC5337972; doi:10.1038/ncomms14113)
Supplement: Supplementary Information — Supplementary figures, supplementary tables, supplementary notes, supplementary methods and supplementary references. [file ncomms14113-s1.pdf]

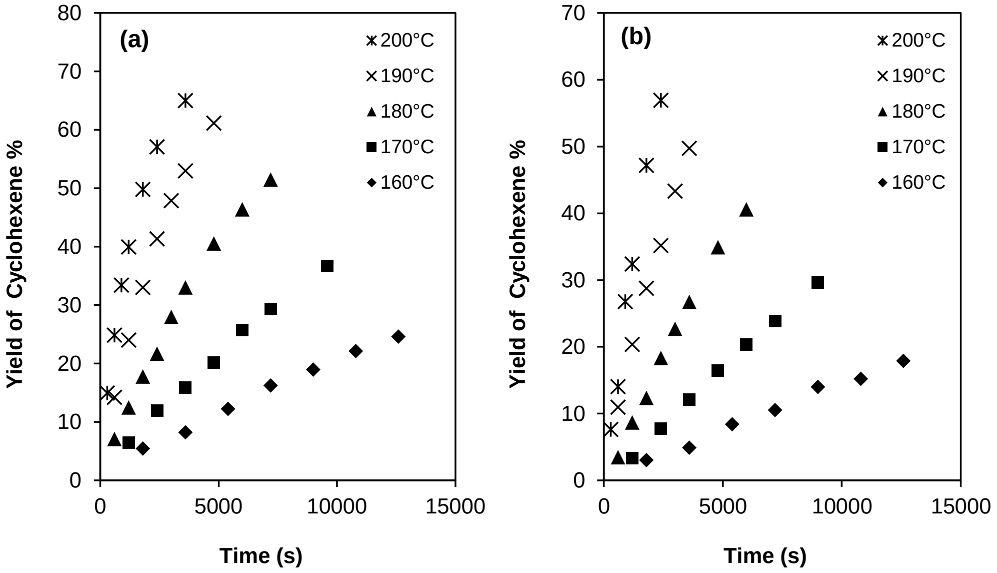

Supplementary Figure 1 | Olefin yield-time plots for H_3PO_4-catalyzed dehydration of cyclohexanol in aqueous phase. (a) 0.32 M cyclohexanol (room temperature, r.t.), (b) 0.90 M cyclohexanol (r.t.). Reaction conditions: cyclohexanol (3.3 or 10.0 g), H_3PO_4 solution (100 mL, 0.02 M at r.t.), 50 bar H_2 (r.t.), stirred at 700 rpm, 160−200 °C.

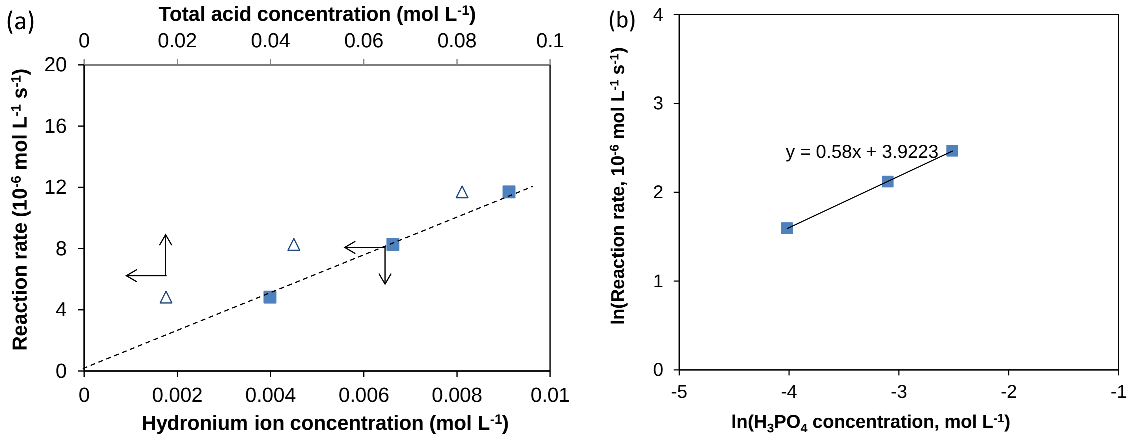

Supplementary Figure 2 | (a) Proportionality of reaction rate to the concentration of hydronium ion and total acid and (b) the reaction order with respect to the concentration of H_3PO_4. The dehydration of cyclohexanol to cyclohexene was carried out at 160 °C in aqueous solutions containing 0.32 M (at r.t.) cyclohexanol and various concentrations of H_3PO_4 (0.02−0.09 M at r.t.). Rates and concentrations in the plots are corrected for solution density at 160 °C. Reaction order with respect to total acid concentration is approximately 0.6 (b). This supports the claim that hydronium ions dissociated from the H_3PO_4 are responsible for the catalytic reaction.

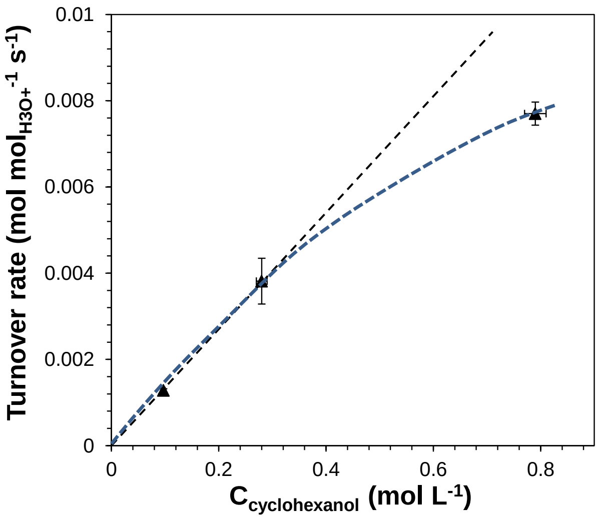

Supplementary Figure 3 | Measurement of the reaction order with respect to cyclohexanol concentration in dilute aqueous H_3PO_4 solutions. Turnover rates (normalized to hydronium ion concentrations) of cyclohexanol dehydration to cyclohexene were measured in aqueous solutions containing 0.02 M (r.t.) H_3PO_4 and various concentrations of cyclohexanol (0.10−0.90 M at r.t.). The uncertainties in the measured rates are < ± 5%. Concentrations of cyclohexanol in the plot have been corrected for vapor phase loss and solution density change at 170 °C. TOFs are based on the corrected concentrations of cyclohexanol and hydronium ions at reaction temperatures (Supplementary Table 11). At low alcohol concentrations (< 0.32 M, r.t.), the dehydration TOF was observed to be first order with respect to the concentration of cyclohexanol. At higher alcohol concentrations (0.90 M, r.t.), the increase in TOF is less than proportional to the increase in the concentration of cyclohexanol.

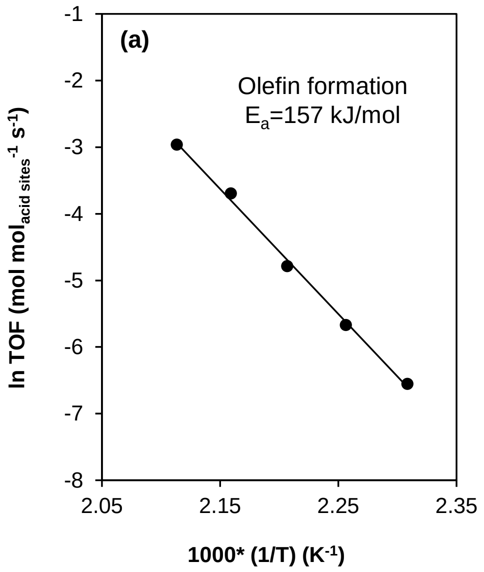

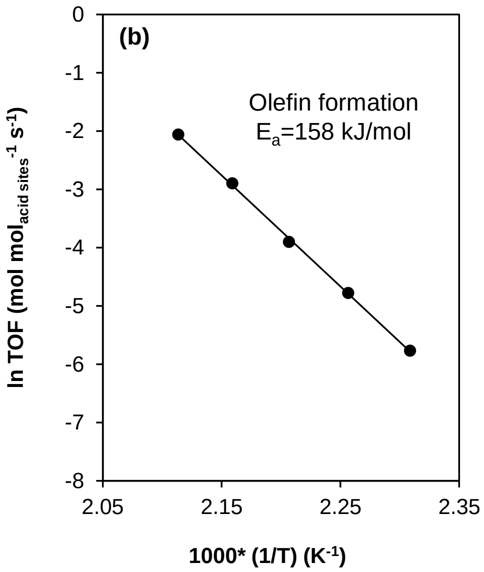

Supplementary Figure 4 | Arrhenius plots for H_3PO_4-catalyzed dehydration of cyclohexanol in aqueous phase. (a) 0.32 M cyclohexanol (r.t.), (b) 0.90 M cyclohexanol (r.t.). Reaction conditions: cyclohexanol (3.3 or 10.0 g), H_3PO_4 solution (100 mL, 0.02 M at r.t.), 50 bar H_2 (r.t.), stirred at 700 rpm, 160−200 °C. TOFs are based on the corrected concentrations of cyclohexanol and hydronium ions at reaction temperatures (Supplementary Table 11).

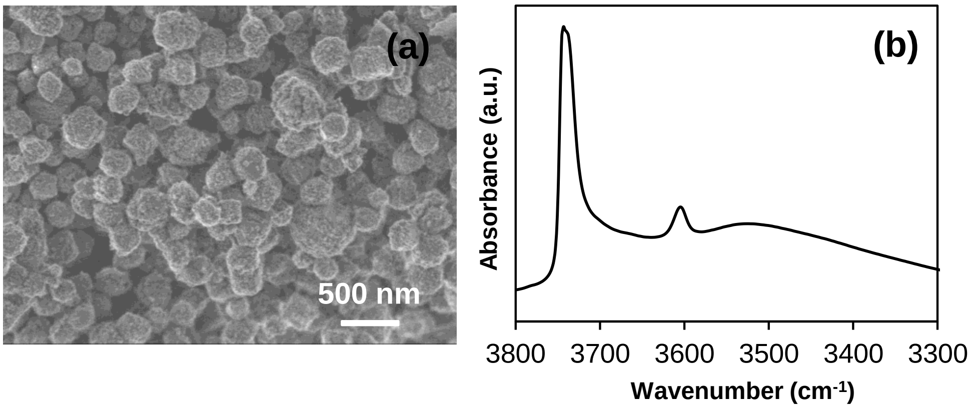

Supplementary Figure 5 | (a) Scanning electron microscopy (SEM) image and (b) the OH-stretching vibration region of the infrared (IR) spectrum of HBEA150. SEM image shows that HBEA150 has particles with rounded corners and average diameters of ~200−300 nm. Two distinct bands of free OH groups were detected; the band at 3740 cm^{-1} is attributed to terminal and internal Si–OH groups (non-acidic), while the band at 3605 cm^{-1} is attributed to the Brønsted-acidic bridging hydroxyl groups associated with Al T sites.

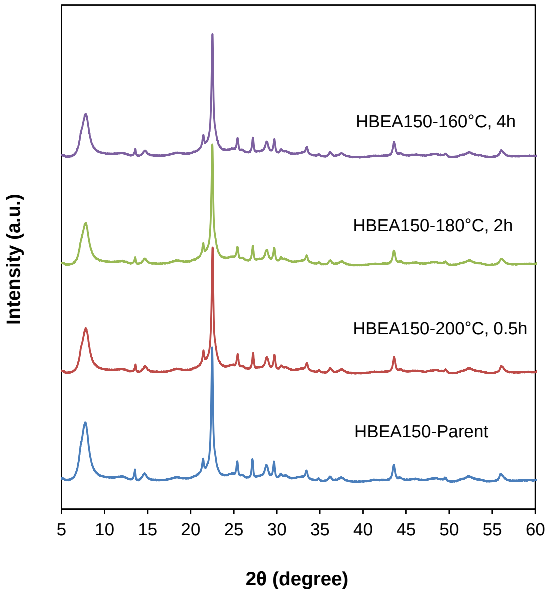

Supplementary Figure 6 | X-ray diffractograms of parent and water treated HBEA150 samples. Sample-specific treatment conditions are reported in the plot. No appreciable changes in XRD patterns were observed after hydrothermal treatment for 0.5−4 h at 160 °C and 0.5−2 h at 180 °C, implying the HBEA150 sample retains structural integrity under conditions typical for catalysis. This was also confirmed by the BET results of these four samples by N_2 adsorption and desorption shown in Supplementary **Table 3.**

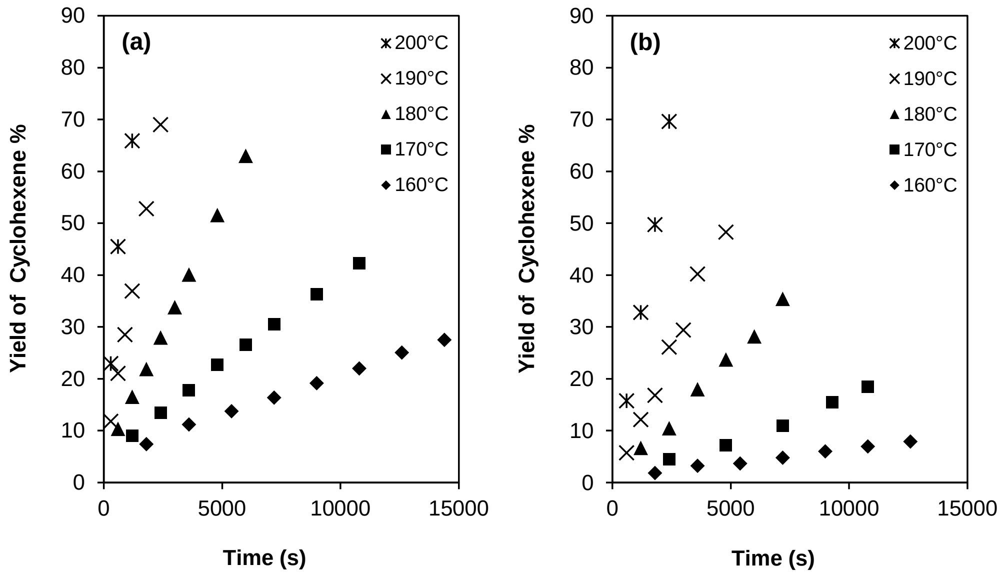

Supplementary Figure 7 | Olefin yield-time plots for HBEA-catalyzed dehydration of cyclohexanol in aqueous phase. (a) 0.32 M cyclohexanol (r.t.), (b) 0.90 M cyclohexanol (r.t.). Reaction conditions: cyclohexanol (3.3 or 10.0 g), HBEA150 (140 mg), water (100 g), 50 bar H_2 (r.t.), stirred at 700 rpm, 160−200 °C.

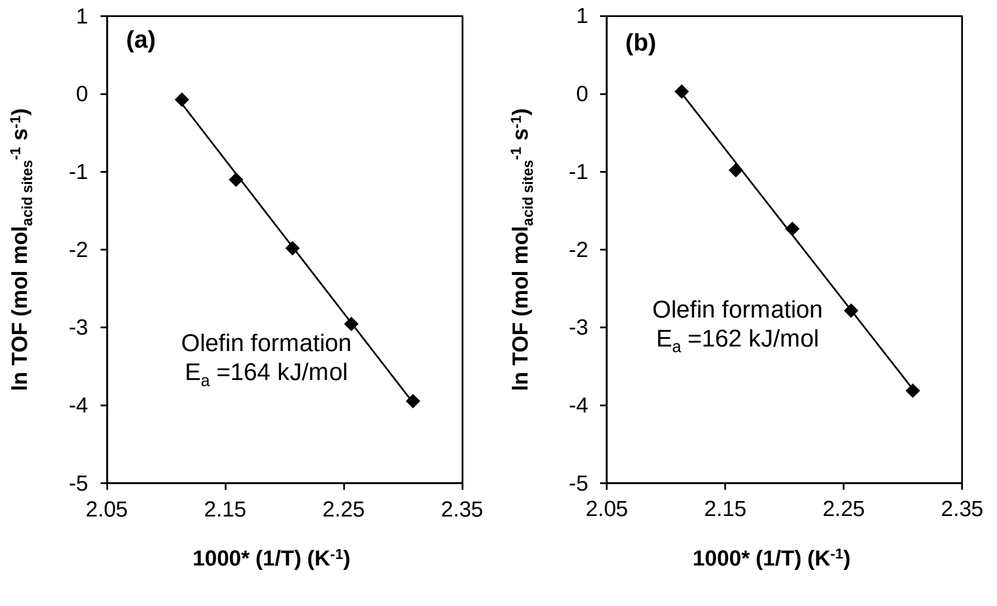

Supplementary Figure 8 | Arrhenius plots for HBEA-catalyzed dehydration of cyclohexanol to cyclohexene in aqueous phase. (a) 0.32 M cyclohexanol (r.t.), (b) 0.90 M cyclohexanol (r.t.). Reaction conditions: cyclohexanol (3.3 or 10.0 g), water (100 g), HBEA150 (140 mg), 50 bar H_2 (r.t.), stirred at 700 rpm, 160−200 °C. TOFs are based on the corrected concentrations of cyclohexanol and hydronium ions at reaction temperatures (Supplementary Table 11).

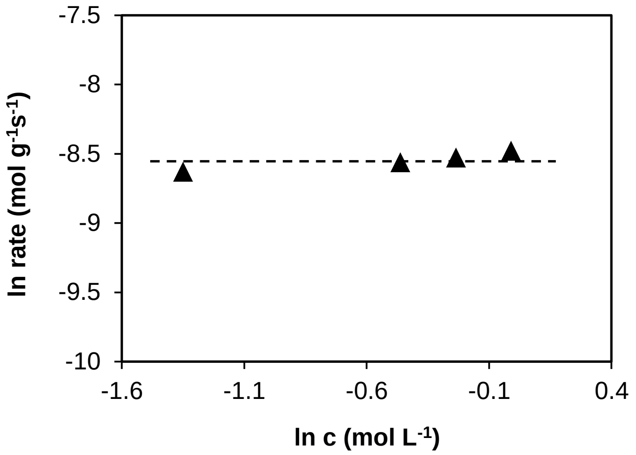

Supplementary Figure 9 | The dependence of the mass-specific reaction rate on the concentration of cyclohexanol for dehydration over HBEA150 in aqueous phase. Reaction conditions: cyclohexanol (3.3 g, 8.0 g, 10.0 g and 12.5 g), HBEA-150 (140 mg), water (100 g), stirred at 700 rpm, 200°C. Aqueous concentrations of cyclohexanol higher than 0.32 M are not possible at room temperature due to the solubility of cyclohexanol in water; at 200 °C, the solutions are monophasic for all of these concentrations (0.32−1.1 M at r.t.). All concentrations in graph have been corrected to 200 °C.

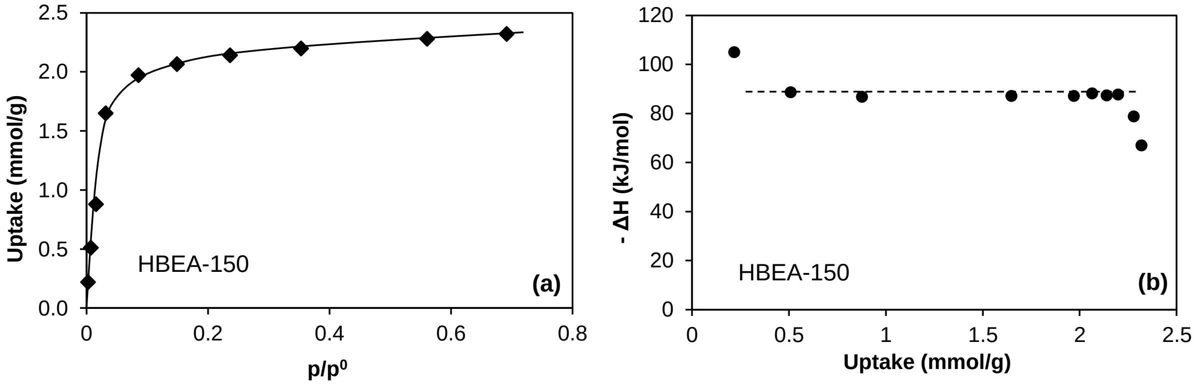

Supplementary Figure 10 | Calorimetric measurements of cyclohexanol adsorption on HBEA150 and siliceous BEA in gas phase. The measurements were performed at 48 °C (p^0 is the saturated vapor pressure of cyclohexanol at 20 °C; p^0 = 1.2 mbar). (a): adsorption isotherms of cyclohexanol on HBEA150; (b): heat of adsorption as function of cyclohexanol uptake on HBEA150. The adsorption isotherm was fitted with a Langmuir adsorption model to derive the adsorption equilibrium constant for HBEA150-a, which was estimated to be 3.4×10^4 bar^{-1} (note: if referenced to the standard state of 1 bar pressure for gas, this constant becomes unitless at the same value).

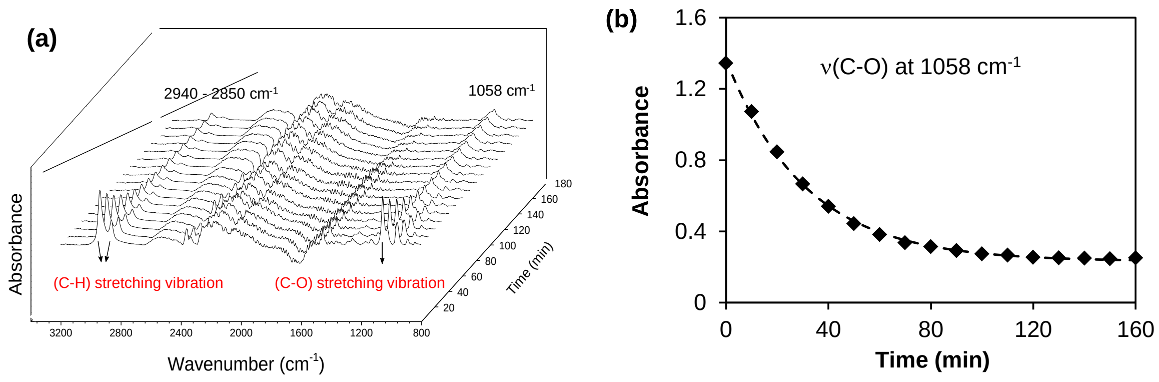

Supplementary Figure 11 | In situ cyclohexanol dehydration in dilute H_3PO_4 solutions monitored with IR. (a) IR spectra acquired during aqueous phase cyclohexanol dehydration catalyzed by H_3PO_4; (b) The measured (markers) and fitted (curve) IR absorbance at 1058 cm^{-1} as a function of residence time. Reaction conditions: 5.0 g cyclohexanol (~0.90 M at r.t.), 50 mL 0.02 M (r.t.) H_3PO_4 in water, 200 °C. 2850–2940 cm^{-1} and 1058 cm^{-1} are attributed to C–H and C–O stretching vibrations from aquesou-phase cyclohexanol, respectively. A reversible first-order equation, derived in ***Supplementary Note 2***, was used for fitting in (b).

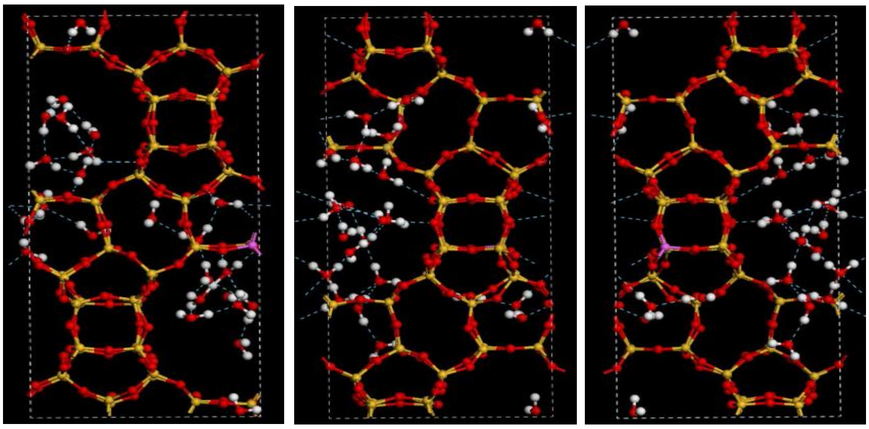

Supplementary Figure 12 | Water Structure inside the pores of HBEA (1 Al per unit cell). Typical hydronium ion cluster containing 8-10 water molecules with a total of 26 water molecules. T = 500 K, AIMD 10 ps.

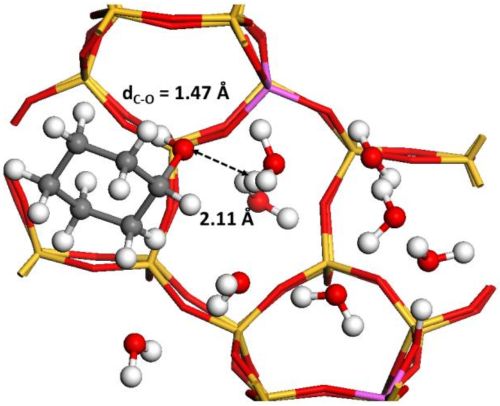

(A)

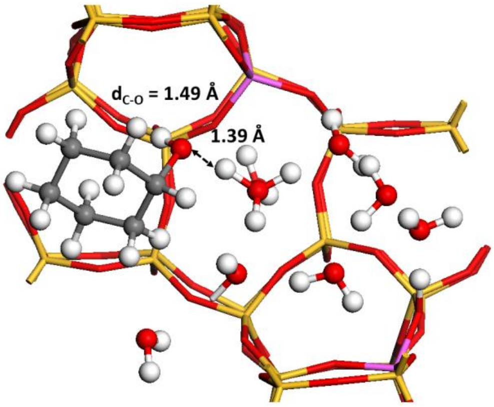

(TS1)

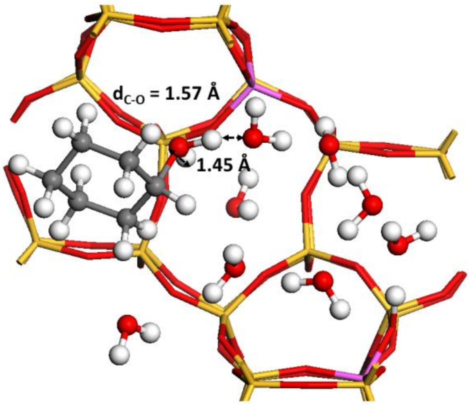

(B)

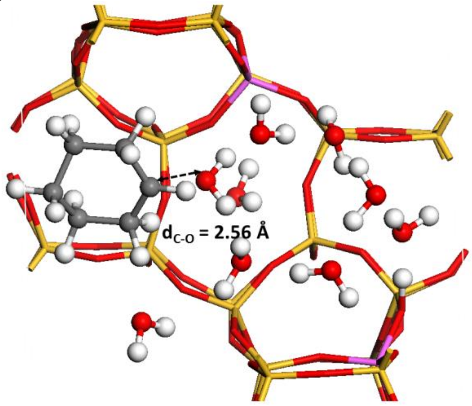

(TS2)

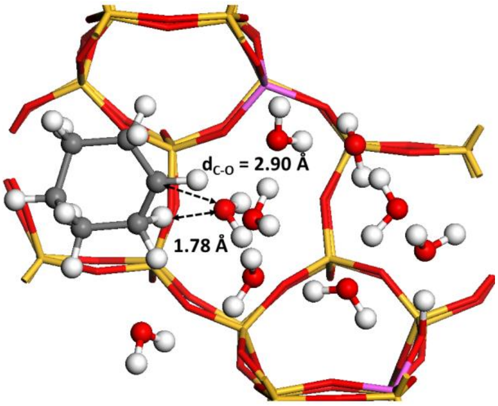

(C)

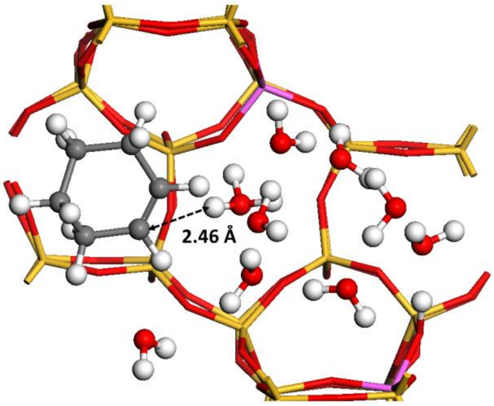

(TS3)

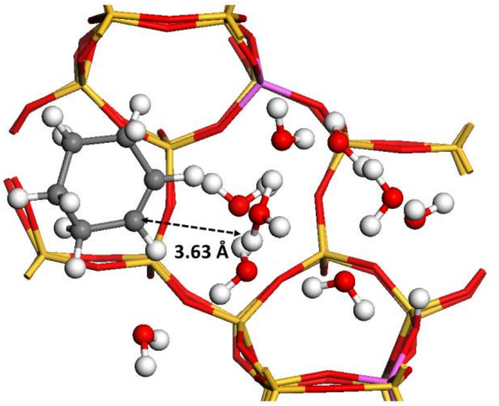

(D)

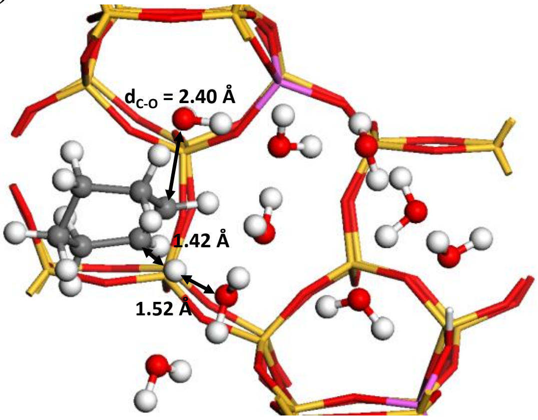

(TS4)

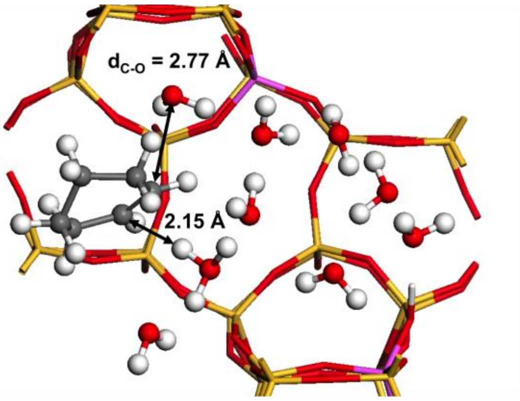

(D')

Supplementary Figure 13 | DFT-optimized structures of reaction intermediates (A–D') and transition states (TS1–TS4) in the aqueous phase cyclohexanol dehydration to cyclohexene in HBEA pore via E1 and E2-like elimination pathways.

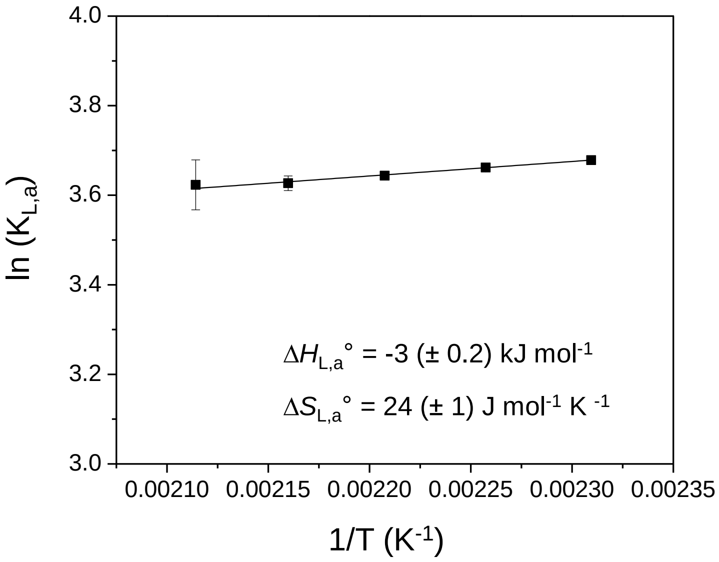

Supplementary Figure 14 | Van't Hoff plot (ln($K_{L,a}$)–(1/T)) to determine the changes in enthalpy ($\Delta H_{L,a}°$) and entropy ($\Delta S_{L,a}°$) for association equilibrium between hydronium ion and cyclohexanol. The detailed calculations of $K_{L,a}$ are shown in ***Supplementary Note 3 and Note 4***, and the values are compiled in ***Supplementary Table 8***.

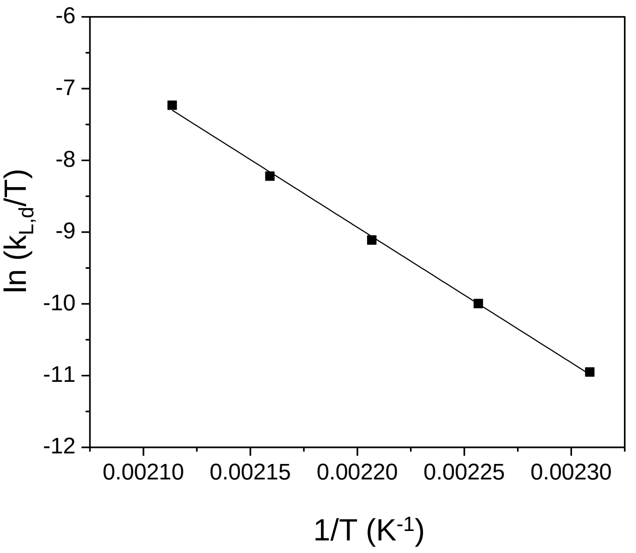

Supplementary Figure 15 | Eyring plot (ln($k_{L,d}$/T)–(1/T)) to determine the enthalpy ($\Delta H^{\circ\ddagger}$) and entropy gained/lost ($\Delta S^{\circ\ddagger}$) required to reach the transition state complex. The detailed method is given in ***Supplementary Note 7***. The values of $k_{L,d}$ are compiled in ***Supplementary Table 8***.

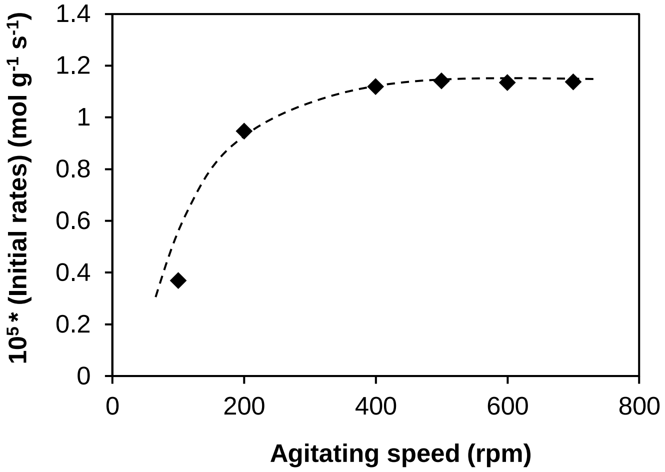

Supplementary Figure 16 | Mass transfer limitation analysis for zeolite-catalyzed dehydration in aqueous phase. Test conditions: 140 mg of HBEA150, 3.3 g of cyclohexanol and 100 g of H_2O, 50 bar H_2 (charged at room temperature), reaction T = 200 °C. Mass transfer limitations do exist when the stirring speed is less than ~ 400 rpm. To exclude mass transfer effects, all kinetic measurements have been performed with a stirring speed of 700 rpm.

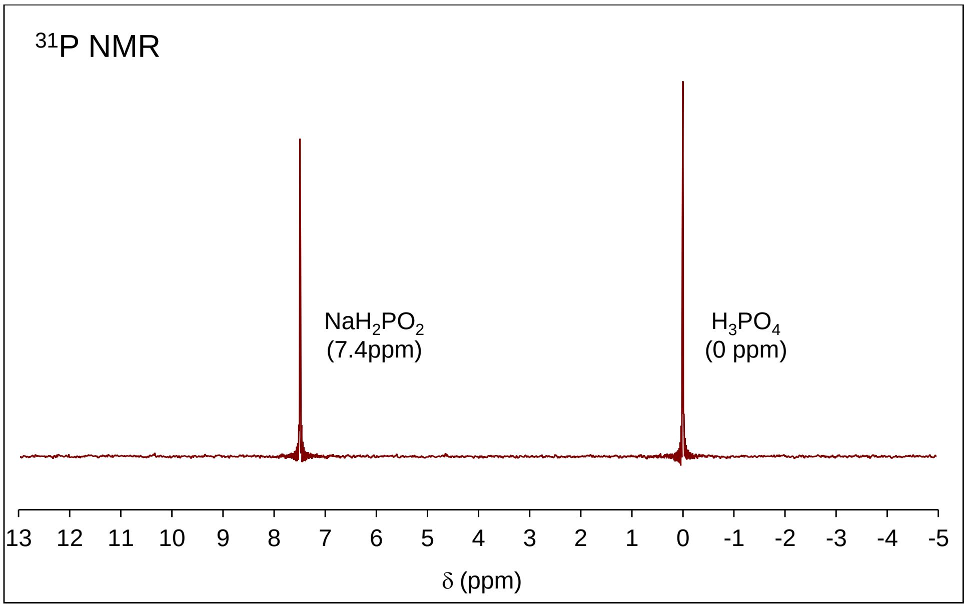

Supplementary Figure 17 | A representative ^{31}P NMR spectrum of a H_3PO_4 solution with NaH_2PO_2 as the internal standard.

Supplementary Table 1 | Dissociation equilibrium constants for the first proton of H_3PO_4 at reaction temperature.[a] *See also* ***Supplementary Note 4.***

Temperature (°C)	$K_{a,1}$ (10^{-4})
160	9.43
170	7.76
180	6.36
190	5.20
200	4.23

[a] The equilibrium constant $K_{a,1}$ for aqueous H_3PO_4 solutions at elevated temperatures is given by $pK_{a,1}$=756.276/T-4.0886+0.012396T.[1] See details in Supplementary Note 4.

Supplementary Table 2 | The textural and acid properties of the studied HBEA zeolite characterized by N_2 adsorption/desorption and IR adsorbed pyridine.

Zeolite	Si/Al ratio[a]	Pore surface area (m^2/g)	Mesopores (m^2/g)	Micropores (m^2/g)	Pore volume (cm^3/g)	Mesopores (cm^3/g)	Micropores (cm^3/g)
HBEA150	71	624	122	502	0.37	0.17	0.20

Zeolite	Acid site concentration [µmol/g]					
	Brønsted	Lewis	Total[b]	Strong Brønsted	Strong Lewis	Strong total
HBEA150	192	41	233	181	24	205

[a] Si/Al ratios are determined from element analysis. [b] Total acid sites are defined as those that retain pyridine after outgassing at 150 °C for 1 h following saturation of the surface by pyridine. [c] Strong acid sites are defined as those that retain pyridine after outgassing at 450 °C for 1 h following saturation of all sites by pyridine.

Supplementary Table 3 | Textural properties of the HBEA150 (parent and hydrothermally treated) samples measured by N_2 adsorption and desorption.

Sample	BET surface area (m^2 g^{-1})			Pore volume (cm^3 g^{-1})		
	Micro	Meso	Total	Micro	Meso	Total
HBEA150 parent	502	122	624	0.20	0.17	0.37
HBEA150 200 °C 0.5 h	373	241	614	0.16	0.34	0.50
HBEA150 180 °C 2 h	376	204	580	0.16	0.29	0.45
HBEA150 160 °C 4 h	406	196	602	0.17	0.28	0.45

Supplementary Table 4 | Reaction rates comparison in the dehydration of cyclohexanol to cyclohexene catalyzed by H_3PO_4, HBEA and their mixture (H_3PO_4+HBEA) at 170°C.[a] ***See also Supplementary Note 6.***

Reaction	Catalyst	Rate ($mol_{cyclohexene}\ s^{-1}$)	Reaction conditions [b]
1	H_3PO_4	3.48×10^{-6} [c]	0.02 M H_3PO_4 (100 mL), 10.0g cyclohexanol, 50bar H_2, 700rpm, 170°C.
2	HBEA150	1.66×10^{-6} [c]	140 mg HBEA-150, 100 mL H_2O, 10.0g cyclohexanol, 50bar H_2, 700rpm, 170°C.
3	H_3PO_4 + HBEA150	6.85×10^{-6}	140 mg HBEA-150, 0.02 M H_3PO_4 (100 mL), 10.0g cyclohexanol, 50bar H_2, 700rpm, 170°C.
4	H_3PO_4 + Si-BEA[d]	3.59×10^{-6}	500 mg Si-BEA, 0.02 M H_3PO_4 (100 mL), 10.0g cyclohexanol, 50bar H_2, 700rpm, 170°C.

[a]The purely siliceous BEA zeolite (Si-BEA) is used as a reference. [b]The concentrations and volumes denoted are based on the density of water at room temperature. [c] Normalizing the rates to the number of hydronium ions will lead to the same TOFs shown in Table 1 in main text. [d] Zeolite Si-BEA is homemade with the physicochemical properties: BAS (0), LAS (0.027 mmol g^{-1}), BET surface area (469 $m^2\ g^{-1}$), total pore volume (0.22 $cm^3\ g^{-1}$).

Supplementary Table 5 | Saturation uptake measured from adsorption isotherms of cyclohexanol from aqueous solutions to zeolite HBEA150. *See also* ***Supplementary Note 1.***

Adsorption temperature (°C)	Saturation uptake (mmol g^{-1})
7	1.75
25	1.57
50	1.54
60	1.47
80	1.31

Supplementary Table 6 | Equilibrium constants, heats of adsorption, and entropy changes for cyclohexanol uptake on HBEA150 zeolite.[a]

Parameter	Gas phase	Aqueous phase
K_{ads}	3.4×10^{4} [b]	3.2×10^{2} [c]
$\Delta H°_{ads}$ (kJ mol^{-1})	–88	–22
$\Delta S°_{ads}$ (J mol^{-1} K^{-1})	–186	–25

[a] Adsorption constants were derived from the slope of the linearized Langmuir isotherm, standard molar enthalpy changes of cyclohexanol adsorption were determined by microcalorimetry and separate isotherm measurements, and standard molar entropy changes were obtained from transition state theory formalism that relates all thermodynamic quantities. Standard states for gas phase molecules, aqueous phase molecules and adsorbed molecules are 1 bar, 1 M, and pore-filling fraction or surface site coverage = 1, respectively; [b] At 48 °C; [c] At 25 °C.

Supplementary Table 7 | Fitted parameters from the least-squares regression of *in situ* kinetics obtained by IR. Reaction conditions: cyclohexanol in H_3PO_4 solution (50 mL, 0.02 M at r.t.), 200 °C, 30 bar H_2 (charged at r.t.), stirred at 900 rpm. The rate expression was derived in ***Supplementary Note 2.***

Parameters	Cyclohexanol concentration	
	0.90 M (r.t.)	0.32 M (r.t.)
k_f (s^{-1})	4.43×10^{-4}	5.77×10^{-4}
$k_{b,eff}$ (s^{-1})[a]	4.34×10^{-5}	8.54×10^{-5}
K_{eq} (dimensionless)[b]	10.2	6.76

[a] Effective first-order reverse reaction constant, $k_{b,eff} = \dfrac{k_b'}{1+\dfrac{1}{K_H RT}\dfrac{V_g}{V_{aq}}}$, as defined in the text.

[b] $K_{eq} = \dfrac{k_f}{\dfrac{k_b'}{1+\dfrac{1}{K_H RT}\dfrac{V_g}{V_{aq}}}}$

Supplementary Table 8 | Equilibrium constants ($K_{L,a}$) for association between hydronium ion and cyclohexanol, the extent of association $\theta_{L,a}$ for two aqueous solutions of cyclohexanol and rate constants ($k_{L,d}$) for H_3PO_4-catalyzed cyclohexanol dehydration at different temperatures. The corrected mole concentrations at high temperatures are compiled in ***Supplementary Table 11***. The enthalpy and entropy changes determined from the Van't Hoff plot of the regressed equilibrium constants ($K_{L,a}$) were found to be -3 kJ mol^{-1} and 24 J mol^{-1} K^{-1}, respectively. *See **Supplementary Figure 14** and **Notes 3** and **4**.*

Temperature (°C)	$K_{L,a}$[a] (dimensionless)	$\theta_{L,a}$		$k_{L,d}$ (s^{-1})
		0.32 M (r.t.)	0.90 M (r.t.)	
160	39.5	1.79×10^{-1}	3.81×10^{-1}	7.61×10^{-3}
170	38.9	1.75×10^{-1}	3.75×10^{-1}	2.02×10^{-2}
180	38.3	1.72×10^{-1}	3.70×10^{-1}	5.01×10^{-2}
190	37.8	1.68×10^{-1}	3.64×10^{-1}	1.25×10^{-1}
200	37.3	1.64×10^{-1}	3.57×10^{-1}	3.42×10^{-1}

[a] Calculated from the Van't Hoff equation.

Supplementary Table 9 | Proton affinities (ΔH_{PA}, for the process A + H^+ → AH^+) of a single water molecule, water clusters and cyclohexanol in vacuum and HBEA.

Species (A)	ΔH_{PA} (kJ mol^{-1})	
	Vacuum	HBEA
H_2O	-702	NA
$(H_2O)_2$	-837	+45
$(H_2O)_3$	-918	-12
$(H_2O)_4$	-930	-31
$(H_2O)_5$	-966	-32
$C_6H_{11}OH$	-787	-25

Supplementary Table 10 | Concentration measured at 25 °C by ^{31}P NMR of H_3PO_4 solutions before and after adding HBEA150 and siliceous BEA. *See also* ***Supplementary Note 6.***

	Concentration of H_3PO_4 in solutions (M)
Reference (0.02 M H_3PO_4)	0.020
Sample A (20-25 mL 0.02 M H_3PO_4 + 1.0 g HBEA150)	~0.0176
Sample B (20-25 mL 0.02 M H_3PO_4 + 1.0 g purely siliceous BEA)	~0.020

Supplementary Table 11 | Concentrations of cyclohexanol, hydronium ion and [ROH]/[H_2O] ratio in the starting reaction mixture at reaction temperatures. I: 3.1 wt% solution; II: 9.1 wt% solution. The densities of both solutions are assumed to change with the temperature as pure water does (d = 0.90, 0.89, 0.88, 0.87 and 0.86 at 160, 170, 180, 190 and 200 °C, respectively).

Temperature (°C)	[ROH] (M)	[ROH]/[H_2O] (10^{-2} M)	[H_3O^+] (10^{-3} M)
160	I: 0.28	I: 0.56	I: 4.0
	II: 0.80	II: 1.59	II: 4.55
170	I: 0.27	I: 0.55	I: 3.6
	II: 0.77	II: 1.57	II: 4.1
180	I: 0.27	I: 0.55	I: 3.3
	II: 0.76	II: 1.55	II: 3.7
190	I: 0.26	I: 0.54	I: 3.0
	II: 0.74	II: 1.54	II: 3.5
200	I: 0.25	I: 0.53	I: 2.7
	II: 0.72	II: 1.51	II: 3.0

Supplementary Table 12 | Henry's law constants of cyclohexanol at 160 and 200 °C (determined in this work), at 170-190 °C (interpolated) and at room temperature (NIST data[2]). Here, K_H = C_{aq}/P_{gas}

Temperature (°C)	K_H (mol L^{-1} bar^{-1})
160	1.45
170	1.06
180	0.79
190	0.59
200	0.45
25	170

Supplementary Note 1: Estimation of adsorption capacity under reaction conditions

At room temperature, the saturation uptake of cyclohexanol was determined to be 1.6 ± 0.1 mmol g_{HBEA}^{-1} (Figure 1). For gas phase adsorption of cyclohexanol, of 2.2 ± 0.2 mmol g_{HBEA}^{-1} was determined (Supplementary Figure 10(a)). Assuming a liquid density (0.962 g cm^{-3}) for the adsorbed cyclohexanol, an uptake of 2.2 mmol g_{HBEA}^{-1} in the absence of water corresponds to 0.23 cm^3 g_{HBEA}^{-1}, comparable to the micropore volume of the sample (Supplementary Table 2). In the presence of water, the saturation uptake of cyclohexanol corresponds to an occupied volume of 0.16 cm^3 g_{HBEA}^{-1}. Subtracting this value from the micropore volume, and if solely attributing this difference (0.04 cm^3 g_{HBEA}^{-1}) to water in the pores, the adsorbed amount of water would be ~2 mmol g_{HBEA}^{-1}.

The adsorption isotherms of cyclohexanol from aqueous solutions onto zeolite HBEA150 have been measured at various temperatures (7–80 °C). Langmuir-type adsorption model, as discussed in the main text, has been applied to fit these measured isotherms to obtain adsorption constant (K_{ads}) and saturation uptake (q_{max}) at each temperature. Detailed results will be reported in a subsequent publication. Important to this work is what we show below regarding the estimation of adsorption capacity under reaction conditions.

It was found that the saturation uptake decreased as adsorption temperature increased (Supplementary Table 5). This decrease in the saturation uptake with increasing adsorption temperature stems from the decrease in density of the adsorbate phase in the micropore (like thermal expansion of a liquid) as a function of temperature. The temperature dependence takes the form:

$$\frac{1}{q_{max}}\frac{dq_{max}}{dT} = -\delta \qquad (1)$$

where δ is the temperature coefficient of expansion.

Plotting measured/regressed saturation adsorption capacity at different temperatures as a function of temperature yielded a slope ($-\delta$) of –0.0037 K^{-1}. Having extrapolating these experimentally determined q_{max} and K_{ads} to reaction temperatures using the same temperature dependence as determined between 7 and 80 °C, we found that the saturation uptake of

cyclohexanol would decrease from 1.05 to 0.92 mmol g_{HBEA}^{-1} at 160–200 °C. Assuming that the remaining micropore volume (total V_{micro} = 0.20 cm^3 g^{-1}) is filled by adsorbed water, the uptake of water in the pore would increase from 3.9 to 4.2 mmol g_{HBEA}^{-1} (compared with 1.8 mmo g_{HBEA}^{-1} at room temperature) with temperature increasing from 160 to 200 °C.

Supplementary Note 2: Derivation of reversible first-order equation for in situ cyclohexanol dehydration in dilute H_3PO_4 solutions monitored with in situ IR (Supplementary Figure 11) and calculations of the related parameters

An illustration of a reversible first-order dehydration reaction in aqueous phase is shown as follows:

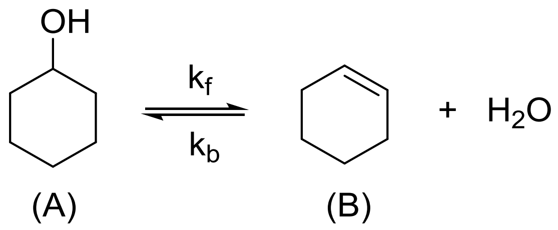

At reaction temperature, however, a significant amount of cyclohexene (B) is distributed into the gas phase. The distribution of B between gas and aqueous phases is defined by Henry's law. It is reasonable to neglect the portion of A that is in the gas phase due to its high boiling point relative to the reaction temperature. The reaction is assumed to occur only in the aqueous solution which contains the catalyst (hydronium ions).

Definitions:

K_H = Henry's law constant in the unit of M/bar

$[A]_{aq}$ = concentration of cyclohexanol in aqueous solution

$[B]_{aq}$ = concentration of cyclohexene in aqueous solution

V_g = volume of reactor headspace

V_{aq} = volume of aqueous solution in the reactor

The total moles ($n_A + n_B$) of A and B is constant in the reactor at different times. Therefore,

$$n_{A,aq,t=0} + n_{B,g,t=0} + n_{B,aq,t=0} = n_{A,aq,t} + n_{B,g,t} + n_{B,aq,t} \tag{2}$$

$$n_{A,aq,t=0} - n_{A,aq,t} = n_{B,g,t} - n_{B,g,t=0} + n_{B,aq,t} - n_{B,aq,t=0} \tag{3}$$

Using Henry's law that applies to the phase distribution for B between gas and solution, we obtain:

$$n_{B,g} = \frac{n_{B,aq}}{V_{aq}K_H}\frac{V_g}{RT} = \frac{n_{B,aq}}{K_H RT}\frac{V_g}{V_{aq}} \tag{4}$$

Substitute into Supplementary Equation (3) and rearrange

$$n_{A,aq,t=0} - n_{A,aq,t} = (n_{B,aq,t} - n_{B,aq,t=0})\left(1 + \frac{1}{K_H RT}\frac{V_g}{V_{aq}}\right) \quad (5)$$

In the same solution, both sides in Supplementary Equation (5) can be divided by V_{aq}, which gives

$$[A]_{aq,t=0} - [A]_{aq,t} = ([B]_{aq,t} - [B]_{aq,t=0})\left(1 + \frac{1}{K_H RT}\frac{V_g}{V_{aq}}\right) \quad (6)$$

$$[B]_{aq,t} = \frac{[A]_{aq,t=0} - [A]_{aq,t}}{1 + \frac{1}{K_H RT}\frac{V_g}{V_{aq}}} + [B]_{aq,t=0} \quad (7)$$

The reaction occurs in the aqueous phase, such that the rate of cyclohexanol consumption is

$$-r = \frac{\partial[A]}{\partial t} = k_f[A]_{aq,t} - k_b[B]_{aq,t}[H_2O] = k_f[A]_{aq,t} - k_b'[B]_{aq,t} \quad (8)$$

where $k_b' = k_b[H_2O]$ as a result of the almost constant molar concentration of water.

Substituting Supplementary Equation (7) into (8) and rearrange

$$-r = \frac{\partial[A]}{\partial t} = \left(k_f + \frac{k_b'}{1 + \frac{1}{K_H RT}\frac{V_g}{V_{aq}}}\right)[A]_{aq,t} - k_b'\left(\frac{[A]_{aq,t=0}}{1 + \frac{1}{K_H RT}\frac{V_g}{V_{aq}}} + [B]_{aq,t=0}\right) \quad (9)$$

At t = ∞, the reaction reaches equilibrium and the net rate drops to zero

$$-r = \frac{\partial[A]}{\partial t} = \left(k_f + \frac{k_b'}{1 + \frac{1}{K_H RT}\frac{V_g}{V_{aq}}}\right)[A]_{aq,t=\infty} - k_b'\left(\frac{[A]_{aq,t=0}}{1 + \frac{1}{K_H RT}\frac{V_g}{V_{aq}}} + [B]_{aq,t=0}\right) \quad (10)$$

Combining Supplementary Equations (9) and (10)

$$-r = \frac{\partial[A]}{\partial t} = \left(k_f + \frac{k_b'}{1 + \frac{1}{K_H RT}\frac{V_g}{V_{aq}}}\right)([A]_{aq,t} - [A]_{aq,t=\infty}) \quad (11)$$

The integration of the above differential equation gives:

$$\int_{[A]_{aq,t=0}}^{[A]_{aq}} \frac{d[A]_{aq}}{[A]_{aq} - [A]_{aq,t=\infty}} = -\left(k_f + \frac{k_b'}{1 + \frac{1}{K_H RT}\frac{V_g}{V_{aq}}}\right)\int_0^t dt \quad (12)$$

$$\ln\frac{[A]_{aq}-[A]_{aq,t=\infty}}{[A]_{aq,t=0}-[A]_{aq,t=\infty}}=-(k_f+\frac{k_b'}{1+\frac{1}{K_H RT}\frac{V_g}{V_{aq}}})t \tag{13}$$

$$([A]_{aq}-[A]_{aq,t=\infty})=([A]_{aq,t=0}-[A]_{aq,t=\infty})\exp[-(k_f+\frac{k_b'}{1+\frac{1}{K_H RT}\frac{V_g}{V_{aq}}})t] \tag{14}$$

Let $[A]_{aq}$ be y (t) and t be x, rearrange Supplementary Equation (14) into

$$y=([A]_{aq,t=0}-[A]_{aq,t=\infty})\exp[-(k_f+\frac{k_b'}{1+\frac{1}{K_H RT}\frac{V_g}{V_{aq}}})x]+[A]_{aq,t=\infty} \tag{15}$$

Supplementary Equation (15) can be used to fit the disappearance of cyclohexanol (IR absorbance or equivalent concentration) in aqueous solution as a function of time.

The React IR results were fitted by this equation. Note that here, the absorbance at 1058 cm^{-1} (C−O stretches of cyclohexanol, reported in Supplementary Figure 12), instead of the concentration of cyclohexanol, is fitted. The physical meanings of other parameters can be referred to in Supplementary Equation (15). Equivalent concentrations of cyclohexanol can be obtained by assuming proportionality between absorbance and concentration in dilute solutions.

The moles of A in the aqueous solution at equilibrium, $n_{A,aq,t=\infty}$, almost equals to the total moles of B ($n_{B,total,t=\infty}$) in both gas ($n_{B,g,t=\infty}$) and solution ($n_{B,aq,t=\infty}$) subtracted from the initial moles of A (no reaction, $n_{A,aq,ini}$). Based on Supplementary Equation (4), together with the above consideration:

$$n_{A,aq,t=\infty}=n_{A,aq,ini}-n_{B,total,t=\infty}=n_{A,aq,ini}-(1+\frac{1}{K_H RT}\frac{V_g}{V_{aq}})n_{B,aq,t=\infty} \tag{16}$$

$$[A]_{aq,t=\infty}=[A]_{aq,ini}-(1+\frac{1}{K_H RT}\frac{V_g}{V_{aq}})[B]_{aq,t=\infty} \tag{17}$$

The starting concentration of [A] in aqueous phase is ca. 0.88 M. At equilibrium, the concentration of A ($[A]_{aq,t=\infty}$) is 0.075 M. According to Supplementary Equation (17), the term of

$$(1+\frac{1}{K_H RT}\frac{V_g}{V_{aq}})[B]_{aq,t=\infty} \text{ is 0.805 M.}$$

At equilibrium, according to Supplementary Equation (8),

$$K_{eq} = \frac{k_f}{\dfrac{k_b'}{1+\dfrac{1}{K_H RT}\dfrac{V_g}{V_{aq}}}} = 0.805 \big/ 0.075 = 10.2$$

Supplementary Table 7 lists the fitted parameters from the least-square regression of *in situ* kinetics based on the time-evolution of IR absorbance for H_3PO_4-catalyzed aqueous phase dehydration of cyclohexanol at 200 °C. The fitting of kinetic results into a reversible first-order kinetics model at two concentrations yields somewhat different forward and reverse rate constants. Overall, the reverse reaction occurs at a much slower rate at conversions < 10 % than the forward reaction. Thus the measured initial rate should primarily reflect the forward dehydration reaction.

Supplementary Note 3: Derivation of rate expression for cyclohexanol dehydration in aqueous phase

A proposed sequence of steps within an E1-type mechanistic framework (with the C–H bond cleavage being kinetically relevant) for aqueous phase dehydration of cyclohexanol catalyzed by H_3PO_4 is shown below:

$$ROH(aq) + H_3O^+(H_2O)_3(aq) \overset{K_{L,a}}{\rightleftharpoons} H_3O^+(H_2O)_2(ROH)(aq) + H_2O(l)$$

$$H_3O^+(H_2O)_2(ROH)(aq) \overset{K_{prot}}{\rightleftharpoons} (H_2O)_3(ROH_2^+)(aq)$$

$$(H_2O)_3(ROH_2^+)(aq) \underset{k_r}{\overset{k_f}{\rightleftharpoons}} (H_2O)_4(R^+)(aq)$$

$$(H_2O)_4(R^+)(aq) \xrightarrow{k_{C\text{-}H}} H_3O^+(H_2O)_3(aq) + R(\text{-}H)$$

Association of the alcohol with hydronium ion and the subsequent protonation is proposed to be sufficiently fast and quasi-equilibrated (a circle on top of a two-way arrow). The hydronium ion is represented as $H^+(H_2O)_4(aq)$, the association complex as $H^+(H_2O)_3ROH(aq)$, the olefin product as R(-H).

It has been demonstrated from isotope experiments (see main text) that the prevalent dehydration mechanism is of E1-type with the C_β–H bond cleavage as the kinetically relevant step, for aqueous phase dehydration of cyclohexanol both in dilute H_3PO_4 and in HBEA. A classical sequence of steps for homogeneous acid catalyzed dehydration is proposed above. A similar sequence should apply to HBEA-catalyzed dehydration in aqueous phase, yet with additional adsorption (from aqueous phase to intrazeolite voids where active sites reside) and desorption steps (from intrazeolite sites to aqueous/gas phases).

Next, we derive the kinetic expression for this mechanistic sequence. We use concentration terms instead of activities for solution species in dilute systems, assuming activity coefficients for the solution species are unity.

For the first step shown above, i.e., association of cyclohexanol with hydronium ion, letting the initial proton concentration be $[H_3O^+]_0$, we have:

$$\frac{[H_2O(l)][H_3O^+(H_2O)_2(ROH)(aq)]}{[ROH(aq)]([H_3O^+]_0 - [H_3O^+(H_2O)_2(ROH)(aq)])} = K_{L,a} \qquad (18)$$

Solving the Supplementary Equation (18) gives:

$$\frac{[H_3O^+(H_2O)_2(ROH)(aq)]}{[H_3O^+]_0} = \frac{K_{L,a}\frac{[ROH(aq)]}{[H_2O(l)]}}{1 + K_{L,a}\frac{[ROH(aq)]}{[H_2O(l)]}} \tag{19}$$

For the second step, proton transfer from water cluster to ROH, we have:

$$\frac{[H_3O^+(H_2O)_2(ROH)(aq)]}{[(H_2O)_3(ROH_2{}^+)(aq)]} = K_{prot} \tag{20}$$

Thus, we have:

$$\frac{[(H_2O)_3(ROH_2{}^+)(aq)]}{[H_3O^+]_0} = \frac{K_{prot}}{1 + K_{prot}}\frac{K_{L,a}\frac{[ROH(aq)]}{[H_2O(l)]}}{1 + K_{L,a}\frac{[ROH(aq)]}{[H_2O(l)]}} \tag{21}$$

For the third step, C–O bond cleavage, applying steady-state assumption to the solvated carbenium-ion intermediate, $(H_2O)_4(R^+)(aq)$, we have:

$$k_f[(H_2O)_3(ROH_2{}^+)(aq)] - k_r[(H_2O)_4(R^+)(aq)] = k_{C-H}[(H_2O)_4(R^+)(aq)] \tag{22}$$

$$[(H_2O)_4(R^+)(aq)] = \frac{k_f[(H_2O)_3(ROH_2{}^+)(aq)]}{k_r + k_{C-H}} \tag{23}$$

The expression for TOF is (equal to that of the fourth step):

$$TOF_L = \frac{k_{C-H}[(H_2O)_4(R^+)(aq)]}{[H_3O^+]_0} \tag{24}$$

Replacing the terms for $[(H_2O)_4(R^+)(aq)]$, we have:

$$TOF_L = \frac{k_f k_{C-H}}{k_r + k_{C-H}}\frac{K_{prot}}{1 + K_{prot}}\frac{K_{L,a}\frac{[ROH(aq)]}{[H_2O(l)]}}{1 + K_{L,a}\frac{[ROH(aq)]}{[H_2O(l)]}} \tag{25}$$

Let $\frac{k_f k_{C-H}}{k_r+k_{C-H}}\frac{K_{prot}}{1+K_{prot}} = k_{\mathrm{L,d}}$, we arrive at the equation (3) shown in the main text:

$$TOF_L = k_{L,d}\frac{K_{L,a}\frac{[ROH(aq)]}{[H_2O(l)]}}{1 + K_{L,a}\frac{[ROH(aq)]}{[H_2O(l)]}}$$

The temperature dependence for $k_{L,d}$, in its current functional form ($\frac{k_f k_{C-H}}{k_r + k_{C-H}} \frac{K_{prot}}{1+K_{prot}}$), is complex. However, considering the DFT estimates for protonation in zeolite, K_{prot} is likely much smaller than 1 such that $\frac{K_{prot}}{1+K_{prot}} \approx K_{prot}$. Moreover, the more rapid microscopic reverse of C–O bond cleavage (k_r) than the C–H bond cleavage ($k_{C\text{-}H}$) would allow $\frac{k_f k_{C-H}}{k_r + k_{C-H}}$ to be approximated as $\frac{k_f k_{C-H}}{k_r} = K_{C-O} k_{C-H}$. Therefore, we have: $k_{L,d} = k_{C-H} K_{C-O} K_{prot}$.

An illustrative energy diagram of aqueous phase cyclohexanol dehydration catalyzed by H_3PO_4, starting with the associated complex formed between cyclohexanol and protonated water cluster (hydronium ion) is given as follows:

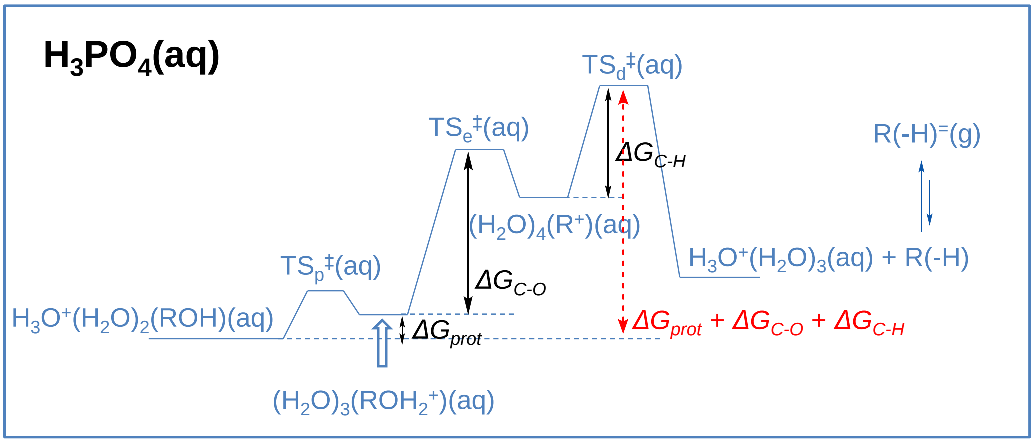

All intermediates and TSs (TS_p, protonation of alcohol; TS_e, C–O bond cleavage leading to elimination of water; TS_d, deprotonation of carbenium ion) are solvated. The schemes do not rigorously reflect the actual relative energy levels of the different states. For $k_{L,d}$ = $k_{C-H} K_{C-O} K_{prot}$, the corresponding changes of enthalpy, entropy and free energy reflect the differences in these parameters between the association complex and the TS for the C_β–H bond cleavage (red broken arrows as shown below). The analysis above applies analogously to zeolite HBEA, with the additional adsorption step.

Supplementary Note 4: Mathematical approach for the determination of hydronium ion concentration, association equilibrium constant and intrinsic rate constant for H_3PO_4-catalyzed dehydration

Since H_3PO_4 is a weak acid with incomplete dissociation of even its first proton (the other two are hardly dissociated) in water at all practical temperatures, the extent of H_3PO_4 dissociation is affected by temperature, total acid concentration, as well as additional equilibria that involve (i.e., consume or produce) any of the species (e.g., H_3PO_4, hydronium ion, anions) that is present in the acid dissociation equilibrium:

$$H_3PO_4 + H_2O = H_2PO_4{}^- + H_3O^+ \tag{26}$$

$$K_{a,1} = \frac{[H_3O^+][H_2PO_4{}^-]}{[H_3PO_4]} \tag{27}$$

The equilibrium constant $K_{a,1}$ is documented for aqueous H_3PO_4 solutions at elevated temperatures, with a functional relation of $pK_{a,1}$=756.276/T-4.0886+0.012396T.[1] The $K_{a,1}$ values for reaction temperatures are listed in Supplementary Table 1.

The hydronium ion is partly associated with cyclohexanol (Supplementary Note 3). Potentially, this additional step with equilibrium constant $K_{L,a}$ would shift the acid dissociation equilibrium as it converts H_3O^+, or $[H^+(H_2O)_4]$, into $[H^+(H_2O)_3ROH]$, as represented in Supplementary Note 3 above. Consequently, $K_{L,a}$ is the key parameter to be solved or regressed; it links the acid dissociation step with known equilibrium constant with the alcohol-hydronium ion association step.

If the concentration of dissociated proton is $[a]$, then $[H^+(H_2O)_3ROH] = [a]*\theta_{L,a}$, $[H_2PO_4^-] = [a]$, $[H^+(H_2O)_4] = [a](1- \theta_{L,a})$, and $[H_3PO_4] = [H_3PO_4]_0 - [a]$ when the two steps reach their respective equilibrium. Thus, we have:

$$K_{a,1} = \frac{[a]\,(1-\theta_{L,a})[a]}{[H_3PO_4]_0 - [a]} \tag{28}$$

where $K_{a,1}$ is known (Supplementary Table 1), $[a] = [H^+(H_2O)_3ROH] + [H^+(H_2O)_4]$, and the extent of alcohol-hydronium ion association, $\theta_{L,a} = \frac{K_{L,a}\frac{[ROH(aq)]}{[H_2O(l)]}}{1+K_{L,a}\frac{[ROH(aq)]}{[H_2O(l)]}}$, is a single-valued function of $K_{L,a}$. The following quadratic equation can be derived:

$$[a] = \frac{-K_{a,1} + \sqrt{(K_{a,1})^2 + 4 \times K_{a,1} \times (1-\theta_{L,a})[H_3PO_4]_0}}{2 \times (1-\theta_{L,a})} \tag{29}$$

From equation (3) in the main text, we find that TOF ratios at two concentrations, 0.32 and 0.90 M (r.t) at which extensive rate data were measured, are equal to the ratios of $\theta_{L,a}$, and would provide another independent functional relation to $K_{L,a}$. TOF is normalized to the summed concentration of $[H^+(H_2O)_3ROH]$ and $[H^+(H_2O)_4]$, that is, $[a]$.

$$\frac{TOF_{L,1}}{TOF_{L,2}} = \frac{\theta_{L,a,1}}{\theta_{L,a,2}} = \frac{\dfrac{K_{L,a}\dfrac{[ROH(aq)]_1}{[H_2O(l)]_1}}{1+K_{L,a}\dfrac{[ROH(aq)]_1}{[H_2O(l)]_1}}}{\dfrac{K_{L,a}\dfrac{[ROH(aq)]_2}{[H_2O(l)]_2}}{1+K_{L,a}\dfrac{[ROH(aq)]_2}{[H_2O(l)]_2}}} \tag{30}$$

While solving $K_{L,a}$ directly from the above functional relationships seems quite challenging, an alternative approach is: 1) give initial guess for $K_{L,a}$ and obtain $\theta_{L,a}$ (recall that $\theta_{L,a} = \frac{K_{L,a}\frac{[ROH(aq)]}{[H_2O(l)]}}{1+K_{L,a}\frac{[ROH(aq)]}{[H_2O(l)]}}$), at each [ROH]/[$H_2O$] ratio; 2) solve for $[a]$ at each [ROH]/[H_2O] ratio from the quadratic equation; 3) use measured volumetric reaction rates and $[a]$ to calculate TOF ratios at the two concentrations; 4) calculate TOF ratios in a Brute Force way that uses the relation that the ratios of TOF are equal to the ratios of $\theta_{L,a}$; 5) regress $K_{L,a}$ to obtain absolute agreement between the TOF ratios determined via steps 1-3 and steps 1,4. The so determined $K_{L,a}$ and $\theta_{L,a}$ are listed in Supplementary Table 8. The concentrations of alcohol, water and hydronium ions (under reaction conditions) used for these calculations are compiled in Supplementary Table 11. The corrections for these concentrations took into account the loss of cyclohexanol and water into the headspace. The Henry's law constants for cyclohexanol in water were experimentally determined at 160 and 200 °C and interpolated to 170, 180 and 190 °C (Supplementary Table 12).

Supplementary Note 5: Association equilibrium constants and intrinsic rate constants for H_3PO_4- and HBEA-catalyzed dehydration

Using measured rate data (H_3PO_4-catalyzed reactions) at the two alcohol concentrations and correcting for solution volume and extent of acid dissociation under reaction conditions, the $K_{L,a}$ and $k_{L,d}$ are determined and compiled in Supplementary Table 8. The enthalpy and entropy changes determined from the Van't Hoff plot of the determined $K_{L,a}$ were found to be -3 kJ mol^{-1} and 24 J mol^{-1} K^{-1}, respectively (Supplementary Figure 14).

From equation (3) in the main text, the rate constants ($k_{L,d}$) were determined from TOF ratios (Table 1 in the main text) and $\theta_{L,a}$ (Supplementary Table 8). Then, the intrinsic activation enthalpy and entropy (reported in Table 4 in the main text) were determined from the Eyring plot of ln($k_{L,d}$/T) as a function of 1/T (Supplementary Figure 15).

For HBEA-catalyzed cyclohexanol dehydration, $\theta_{z,a}$ is likely close to 1, as a result of the [ROH]/[H_2O] in the pore being 0.25 (5 and 20/u.c. for cyclohexanol and water, respectively, at reaction conditions); almost every hydronium ion in the pore is associated with cyclohexanol. In this case, $TOF_z \approx k_{z,d}$, and the ratio of $k_{z,d}/k_{L,d}$ was determined to be 2.7 ± 0.2, indicating that the intrinsic rate constants for cyclohexanol dehydration in HBEA are substantially higher than in aqueous solution.

Supplementary Note 6: Dehydration experiments using mixtures of H_3PO_4 and zeolite

The observed rate with a mixture of H_3PO_4 and HBEA was higher than the sum of rates obtained with the individual acids (see Supplementary Table 4), presumably as a result of phosphoric acid being adsorbed in the pore. On the contrary, no increase in dehydration rate was observed using the mixture of siliceous BEA (Si-BEA) and H_3PO_4. We attribute this to the well-known high hydrophobicity of the all-siliceous BEA that prevents an appreciable amount of water and H_3PO_4 from entering the pore.

To confirm our speculations, H_3PO_4 uptake on HBEA150 and siliceous BEA (Si-BEA) was measured at 25 °C by ^{31}P NMR spectroscopy. We observed significant H_3PO_4 adsorption on zeolite HBEA150 (56 μmol g^{-1}) but found no measurable uptake by Si-BEA (Supplementary Table 10). This proves that H_3PO_4 could diffuse into the pores of HBEA150, but could not get into Si-BEA. However, because the NMR measurement was not performed under the reaction conditions (170 °C, in the presence of cyclohexanol), and because the extent of H_3PO_4 dissociation in the zeolite pore is not known, it is currently not possible to establish a quantitative relation between the increase in the number of acidic species in the pore and the activity enhancement with the combination of H_3PO_4 and HBEA150.

Supplementary Note 7: Calculation of activation enthalpies and entropies based on transition state theory formalism

Transition state theory (TST) assumes that a hypothetical transition state (activated complex) exists between reactants and products during a chemical reaction and that a quasi-equilibrium is established between the reactant and the TS. According to the Erying equation, if the rate constant has been experimentally determined, the theory can be used to calculate the Gibbs free energy, activation enthalpy and entropy. The results are compiled in Table 4 of the main text. The approach is briefly summarized below:

$$k_{rxn} = \frac{k_B T}{h} e^{\Delta S^{\ddagger}/R} e^{-\Delta H^{\ddagger}/RT} \tag{31}$$

Rearrange the Supplementary Equation (31) into the logarithmic form:

$$\ln\left(\frac{k_{rxn}}{T}\right) = \left(\ln\frac{k_B}{h} + \frac{\Delta S^{\ddagger}}{R}\right) - \frac{\Delta H^{\ddagger}}{R}\left(\frac{1}{T}\right) \tag{32}$$

Thus, the enthalpy required ($\Delta H^{\ddagger}$) and entropy gained/lost ($\Delta S^{\ddagger}$) to reach the transition state complex can be determined using Eyring plots (ln(k/T)–(1/T)), see Supplementary Equation (32).

Supplementary Note 8: Error analysis for kinetic parameters

The standard error in $\Delta G^{\circ\ddagger}$ was estimated from the quantities obtained from the sum of squares of residuals that is determined by the regression analysis of the intrinsic rate constant. Specifically, for zeolite, $TOF_z = k_z$, according to the Eyring equation,

$$\Delta G^{\circ\ddagger} = RT\ln(k_B/h) - RT\ln(TOF_z/T) \tag{33}$$

Here, $RT\ln(k_B/h)$ is a constant, so for $\Delta G^{\circ\ddagger}$, the only error source is $\Delta\ln(TOF_z/T)$. Then, we have $\Delta\Delta G^{\circ\ddagger} = RT*\Delta\ln(TOF_Z/T)$. $\Delta H^{\circ\ddagger}$ and $\Delta S^{\circ\ddagger}$ as they are derived from the slope and intercept of the Eyring plot shown in Supplementary Equation (32). For $\Delta H^{\circ\ddagger}$ and $\Delta S^{\circ\ddagger}$, the probability density of the normal distribution is given by the equation:

$$f(x) = \frac{1}{\sqrt{2\sigma^2\pi}}\exp[-\frac{(x-\mu)^2}{2\sigma^2}] \tag{34}$$

μ is the mean or expectation of the distribution for values of $\Delta H^{\circ\ddagger}$ and $\Delta S^{\circ\ddagger}$, while σ is the standard deviation. Then we have the normal distribution for $\Delta H^{\circ\ddagger}$ and $\Delta S^{\circ\ddagger}$.

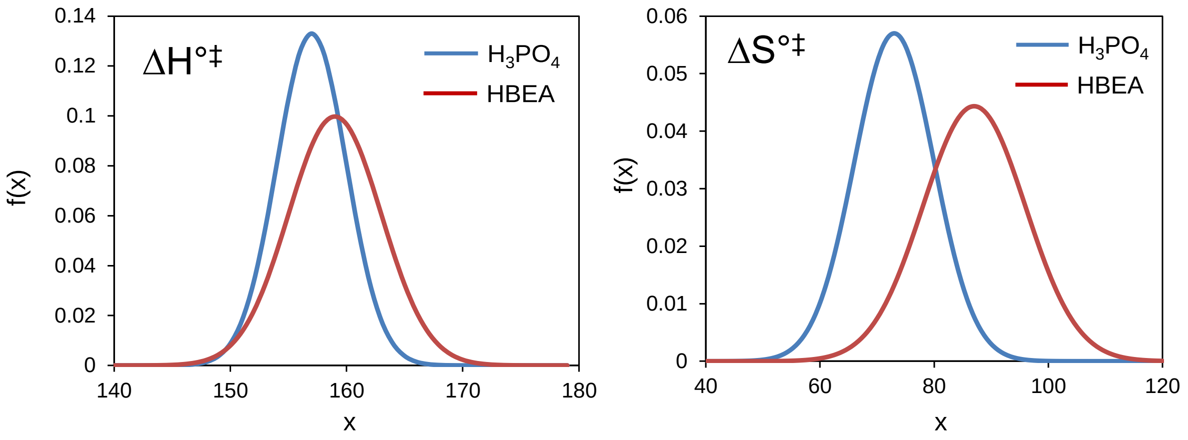

According to the probability density function, we can compare the $\Delta H^{\circ\ddagger}$ and $\Delta S^{\circ\ddagger}$ in H_3PO_4- and HBEA-catalyzed cyclohexanol dehydration, as shown below:

Probability	$\Delta H^{\circ\ddagger}$	$\Delta S^{\circ\ddagger}$
HBEA>H_3PO_4	0.69	0.90
HBEA=H_3PO_4	0.07	0.02
HBEA<H_3PO_4	0.24	0.08

It can be seen that in terms of $\Delta H^{\circ\ddagger}$, 157 (± 3) kJ mol^{-1} for H_3PO_4-catalyzed dehydration is nearly the same as 159 (± 4) kJ mol^{-1} for HBEA-catalyzed dehydration, while for $\Delta S^{\circ\ddagger}$, the difference is statistically significant, i.e., the $\Delta S^{\circ\ddagger}$ in HBEA-catalyzed reaction is larger than that in H_3PO_4-catalyzed reaction with a 90% probability.

Supplementary Methods

The Si and Al contents in the zeolite samples were measured by atomic absorption spectroscopy (AAS) on a UNICAM 939 AA–Spectrometer.

The BET specific surface area and pore volume (BJH method) were determined after activation in vacuum at 200 °C for 2 h followed by nitrogen adsorption on a PMI automatic Sorptometer.

The scanning electron microscopy (SEM) images were recorded on a JEOL 500 scanning electron microscope (accelerating voltage 25 kV). The samples were prepared by depositing a drop of an ultrasonicated methanol suspension of the solid material onto a carbon-coated Cu grid. The dry samples were gold–coated prior to imaging.

XRD patterns were collected using a Philips X'Pert Pro System, with Cu-Kα radiation source operating at 45 kV and 40 mA. The sample was measured with a scanning rate of 0.02° s^{-1} in the 5–70° 2θ-range.

The infrared (IR) spectra of adsorbed pyridine were recorded with a Perkin–Elmer 2000 spectrometer at a resolution of 4 cm^{-1}. The catalyst samples were prepared as self–supporting wafers and activated in vacuum ($p = 10^{-6}$ mbar) at 450 °C for 1 h at a heating rate of 10 °C min^{-1}. After cooling to 150 °C, the sample was equilibrated with 0.1 mbar pyridine for 0.5 h followed by outgassing for 1 h and the acquisition of the spectrum. Finally, desorption program (up to 450 °C with 10 °C min^{-1} and 0.5 h at 450 °C) was initiated and the spectra were recorded until equilibrium was achieved. The concentrations of BAS and Lewis acid sites (LAS) are quantified using the integrated areas of peaks at 1540 cm^{-1} and 1450 cm^{-1}, respectively. The number of pyridine molecules retained after evacuation at 150 and 450 °C were used to determine the concentrations of total and strong acid sites, respectively. For calibration of the method, a standard (Zeolite HZSM-5 with Si/Al = 45, acid site concentration = 360 µmol g^{-1}) was used. For quantification, molar integral extinction coefficients of 0.73 cm μmol^{-1} and 0.96 cm μmol^{-1} were used for Brønsted and Lewis acid sites, respectively.

An in situ time-resolved IR study was conducted in a Parr reactor of a similar head-space-to-liquid volume ratio and feed composition at 200 °C, in order to confirm the validity of the ex-situ GC analyses of reaction kinetics. Measurements were performed using a React IR 1000

spectrometer (Mettler Toledo) connected to a 100 mL PARR Hastelloy autoclave. A diamond window in the autoclave allowed collection of the in situ liquid IR spectra. First, a background is collected for the system containing 50 mL 0.02 M H_3PO_4 solution at 200 °C in the presence of 3.0 MPa H_2. The reactor is then cooled to ambient temperature and 5.0 g cyclohexanol (~0.90 M, fully miscible with water at 200 °C) is added, the autoclave is flushed with H_2 and pressurized 3.0 MPa H_2. IR spectra are collected every 10 min for 240 min at 200 °C with a stirring speed of 900 rpm.

Cyclohexanol dehydration reactions catalyzed by the mixture of HBEA150/siliceous BEA and H_3PO_4 were performed at 170 °C using a 300 mL Hastelloy PARR reactor. 140 mg HBEA150 or 500mg siliceous BEA together with 10.0 g cyclohexanol and 100 mL 0.02 M aqueous H_3PO_4 (Sigma Aldrich, ≥99.999% trace metals basis) were sealed in the reactor. The experimental protocol was identical to that described in the Methods section (main text). The results are compiled in Supplementary Table 4.

Gas-phase calorimetric and gravimetric measurements were performed at 48 °C on a Setaram TGA-DSC 111 microbalance attached to a UHV system. The catalyst was first pressed into wafers, subsequently crushed in small particles and then charged into a crucible with the mass between 13 to 22 mg. Before measurement, the sample was activated at 450 °C for 1 h with a heating ramp of 10 °C min^{-1} under vacuum ($p < 10^{-6}$ bar). Cyclohexanol vapor was introduced into the closed system in small pressure steps from 10^{-3} to 0.8 mbar, allowing sufficient time to reach adsorption equilibration. The weight increase and heat flux were monitored during pressure equilibration with cyclohexanol. The heats of adsorption were obtained by integration of the recorded heat flux signal observed during stepwise increase of the cyclohexanol pressure.

1.0 g HBEA150 was added into ~20-25 mL aqueous H_3PO_4 (0.02 M), allowed to equilibrate in the oven at 25 °C for 24-48 h, and centrifuged to retain the solution (Sample A). The same experiment was performed with a purely siliceous BEA (Sample B). The reference was the same H_3PO_4 solution without adding HBEA150. Samples for ^{31}P NMR measurements were prepared by mixing 300 µL of H_3PO_4 solution (Solution A or B, or reference) and 200 µL of D_2O (99.9 atom % D, Sigma-Aldrich), and then 300 µL of 0.02 M NaH_2PO_2 (Sigma Aldrich, ≥99%; dissolved in water) was added as the internal standard for quantification. 600 µL of such a mixture was placed in an NMR sample tube and the measurement was performed on a Bruker AVHD 300 spectrometer with a deuterium lock resonance. ^{31}P free induction decays (FIDs) were collected at 12149.5 Hz by using 11.25 µs pulses and 36 s relaxation delays (with

decoupling). Both sample and reference solutions were prepared and measured for three times. A spectrum for the reference solution is shown as Supplementary Figure 17 and the quantitative results are compiled in Supplementary Table 10.

Supplementary References

(1) Rudolph, W. W. Raman-Spectroscopic Measurements of the First Dissociation Constant of Aqueous Phosphoric Acid Solution from 5 to 301 °C. *J Solution Chem* **41**, 630-645, (2012).

(2) Http://webbook.nist.gov/cgi/cbook.cgi?ID=C108930&Mask=10#Solubility
